# Transcriptional and epigenetic decoding of the microglial aging process

**DOI:** 10.1038/s43587-023-00479-x

**Published:** 2023-09-11

**Authors:** Xiaoyu Li, Yuxin Li, Yuxiao Jin, Yuheng Zhang, Jingchuan Wu, Zhen Xu, Yubin Huang, Lin Cai, Shuai Gao, Taohui Liu, Fanzhuo Zeng, Yafei Wang, Wenxu Wang, Ti-Fei Yuan, Hengli Tian, Yousheng Shu, Feifan Guo, Wei Lu, Ying Mao, Xifan Mei, Yanxia Rao, Bo Peng

**Affiliations:** 1 Department of Neurosurgery, Jinshan Hospital, Institute for Translational Brain Research, State Key Laboratory of Medical Neurobiology, MOE Frontiers Center for Brain Science, Innovative Center for New Drug Development of Immune Inflammatory Diseases, Ministry of Education, Fudan University, Shanghai, China; 2 Department of Neurology, Zhongshan Hospital, Department of Laboratory Animal Science, MOE Frontiers Center for Brain Science, Fudan University, Shanghai, China; 3 Department of Neurosurgery, Huashan Hospital, Fudan University, Shanghai, China; 4 Shenzhen Institute of Advanced Technology, Chinese Academy of Sciences, Shenzhen, China; 5 Department of Neurology, Shanghai Sixth People’s Hospital Affiliated to Shanghai Jiao Tong University School of Medicine, Shanghai, China; 6 Department of Orthopedics, The First Affiliated Hospital of Jinzhou Medical University, Jinzhou, China; 7 Shanghai Key Laboratory of Psychotic Disorders, Shanghai Mental Health Center, Shanghai Jiao Tong University School of Medicine, Shanghai, China; 8 Co-Innovation Center of Neurodegeneration, Nantong University, Nantong, China

**Keywords:** Microglia, Neuroimmunology, Ageing

## Abstract

As important immune cells, microglia undergo a series of alterations during aging that increase the susceptibility to brain dysfunctions. However, the longitudinal characteristics of microglia remain poorly understood. In this study, we mapped the transcriptional and epigenetic profiles of microglia from 3- to 24-month-old mice. We first discovered unexpected sex differences and identified age-dependent microglia (ADEM) genes during the aging process. We then compared the features of aging and reactivity in female microglia at single-cell resolution and epigenetic level. To dissect functions of aged microglia excluding the influence from other aged brain cells, we established an accelerated microglial turnover model without directly affecting other brain cells. By this model, we achieved aged-like microglia in non-aged brains and confirmed that aged-like microglia per se contribute to cognitive decline. Collectively, our work provides a comprehensive resource for decoding the aging process of microglia, shedding light on how microglia maintain brain functions.

## Abstract (web summary)

Li et al. provide a transcriptional and epigenetic characterization of microglia in aging mice brain by developing a three-round depletion–repopulation (3xDR) model to study aged microglia in non-aged brain, giving insights into the molecular mechanism underlying microglia aging.

## Main

Microglia are resident immune cells in the central nervous system (CNS) that play essential roles in the maintenance of brain homeostasis from the embryonic brain rudiment to the aged brain^1,2^. In recent decades, non-immune functions of microglia have been focused on spine pruning^3^, neuronal excitability modulation^4^ and blood–brain barrier (BBB) regulation^5^. With technological advances at single-cell resolution, researchers found that brain microglia are composed of heterogeneous populations among organisms with different lifespans, sexes and species^6–9^. It also boosted the identification of microglial markers, regulatory factors, pathways and gene landscapes in the context of development, homeostasis and disease. Dissecting the transcriptional and epigenetic characteristics of microglia is critical for understanding how the CNS maintains the homeostasis.

Age is among the top risk factors for brain disorders. Microglial cell aging contributes to brain dysfunction. On the one hand, microglia exhibit an innate immune memory and can be primed by their milieu. Even a mild challenge in early lifespan can induce lifelong susceptibility to a second stimulus, leading to exaggerated immune responses^10,11^. Primed microglia with exaggerated immune responses exhibit a neurotoxic phenotype during brain aging and neurodegeneration^12,13^. Microglia in the aged brain are more likely to be primed during early events and accelerate the aggregation of amyloid-beta (Aβ) in Alzheimer’s disease (AD)^14,15^. In addition, microglia primed by α-synuclein overproduce pro-inflammatory factors and reactive oxygen species (ROS)^16,17^, which contribute to prolonged neuroinflammation and cause the dopamine neuron impairment in Parkinson’s disease (PD)^18,19^. Previous studies suggested that anti-inflammation therapy based on non-steroidal anti-inflammatory drugs (NSAIDs) may ameliorate the pathology of neurodegeneration, including AD and PD^18,20–24^, although the results are controversial^25–27^.

On the other hand, as immune cells, microglia are sentries surveilling and defending the CNS. Microglia show declined functions of surveillance and phagocytosis in aged animals^28^. The aged brain produces more myelin debris than the young brain. As the professional phagocyte in the CNS, microglial dysfunction in aged brains leads to the accumulation of insoluble myelin debris in microglia, such as lipofuscin granules and lipid droplets. The undegradable debris in turn exaggerates microglial dysfunction and myelin degeneration, contributing to cognitive impairment and brain aging^8,29,30^. Aged microglia are associated with α-synuclein aggregation in neurons, suggesting a negative impact on other brain cells^31,32^. Moreover, dysfunctional microglia exacerbate neurodegeneration. Genome-wide association studies identified mutations in microglia (for example, *TREM2* and *APOE*) as major risk factors in late-onset AD^33^. It has been well documented that *Trem2* deficiency in microglia also increases the risks of tauopathies, PD, Nasu–Hakola disease and frontotemporal dementia^34,35^. In contrast, the loss of TREM2 exerts both beneficial and detrimental effects in diverse contexts of tauopathy^36–40^. Correcting the *Trem2* deficiency by microglia replacement^41^ might be beneficial for treating these diseases. Collectively, microglia in the aged brain are associated with the etiology of multiple brain disorders. Nonetheless, how microglia age at the transcriptional and epigenetic levels is not fully understood.

In this study, we systematically investigated microglial transcriptomes from young, middle-aged and aged mouse brains of both sexes. We found that female microglia exhibited a progressive aging process with young, middle-aged and aged stages. In contrast, male microglia did not show the stepwise transition or intermediate middle-aged stage during the aging process. We then dissected the transcriptional dynamics by single-cell RNA sequencing (scRNA-seq) and assessed genome-wide chromatin accessibility by assay for transposase-accessible chromatin using sequencing (ATAC-seq) across young, middle-aged and aged stages in female mice to dissect the molecular mechanisms regulating microglial aging. As surveillance immune cells in the CNS, microglia are sensitive to the microenvironment. To understand how microglia respond to their milieu, we analyzed the transcriptional and epigenetic alterations upon lipopolysaccharide (LPS) challenge at all three stages of female microglia. We further investigated the potential communication of microglia with other brain cells at different ages.

When researchers study a specific aged cell type, what they actually obtain is the summation of all cell types. Different aged cell types influence each other, and the phenotype of a specific cell is shaped by other cell types. Similarly, when studying the contribution of a particular aged cell to a specific function, it is practically the summation of all aged cell types. Microglia and their milieu are tightly related. When a microglial cell ages, other brain cells also advance in age^42^. It is difficult to dissect the functions of aged microglia excluding the contributions from other aged brain cells. Microglia are long-lived myeloid cells with a relatively low turnover rate^43,44^. Colony-stimulating factor 1 receptor (CSF1R) is exclusively expressed in myeloid cells of the brain. The inhibition of CSF1R by PLX5622 ablates the majority of brain microglia without directly affecting other non-myeloid cells. Once the CSF1R inhibition is removed, residual microglia rapidly repopulate the whole brain by proliferation^45^. After three-round depletion–repopulation (3xDR), each microglial cell has proliferated 20+ times. 3xDR shortened the telomere length of microglia, similar to aged microglia^46,47^. Because the cumulative number of microglial divisions is around 20 cycles in the 2-month-old mouse^48^, each microglial cell proliferates about 40 times, close to the Hayflick limit. Our results reveal that 3xDR microglia exhibit an aged (or aged-like) phenotype in the non-aged brain. 3xDR thus allows researchers to decode the independent functions of aged-like microglia, excluding contributions from other aged cells. By using 3xDR, we revealed that aged-like microglia per se contribute to cognitive decline and myelin impairment.

In summary, this study aims to achieve a deep understanding of age-related changes in microglia, decipher the molecular mechanism underlying microglial cell aging and correlate microglial behaviors with brain aging. To disseminate our data to the community, we generated an interactive website for searching the data (http://www.microgliatlas.com).

## Results

### Characteristics of age-dependent genes in microglia across the adult lifespan

**Fig. 1:**
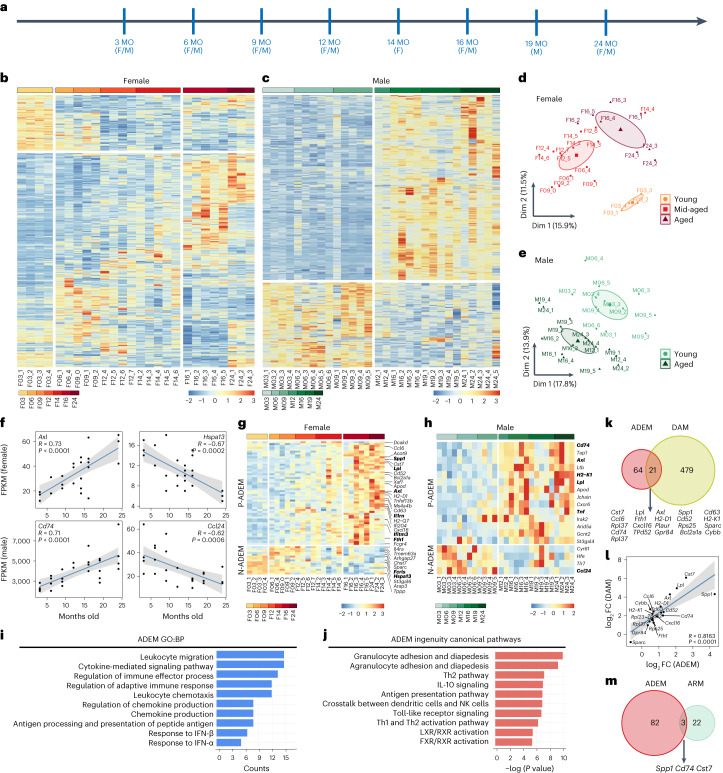
Transcriptional profiling by bulk RNA-seq to investigate the sex-dependent microglial aging process and ADEM gene set. **a**, Scheme of timepoints for microglia bulk RNA-seq analysis. Microglia were collected by FACS from C57BL/6J female and male mouse brains. **b**,**c**, Heat maps of microglial DEGs during the aging process in female (**b**) and male (**c**) mice. Cells are colored according to the *z*-score, and stages are separated by gap. **d**,**e**, PCA plots of microglial phenotypes during the aging process in female (**d**) and male (**e**) mice. **f**, Scatter plot showing linear regression of representative genes correlated with the aging process. Gray shading represents the 95% confidence interval, and Pearson’s correlation coefficients and *P* values are shown. **g**,**h**, Heat maps of ADEM genes during the aging process in female (**g**) and male (**h**) mice. Cells are colored according to the *z*-score, and stages are separated by gap. **i**, Top 10 significantly enriched BPs of ADEM genes annotated by GO (*q* < 0.05). **j**, Top 10 significantly enriched canonical pathways of ADEM genes annotated by IPA (*q* < 0.05). **k**, Venn diagram revealing 21 genes overlapping between the ADEM and DAM gene sets. Genes in both ADEM and DAM gene sets are listed below. **l**, Scatter plot showing linear regression of log_2_FC of overlapping genes between ADEM and DAM gene sets, revealing that they are positively correlated. Gray shading represents the 95% confidence interval, and Pearson’s correlation coefficient and *P* value are shown at the bottom. **m**, Venn plot revealing that only three genes are overlapping between ADEM and ARM gene sets. Genes in both ADEM and ARM gene sets are listed below. *n* = 2–6 mice for each group. BP, biological process; F, female; F03_1, 3-month-old female, biological replicate 1; M03_1, 3-month-old male, biological replicate 1, and so on in a similar fashion; M, male; MO, months old; Dim, dimension. Source data

To obtain deep insights into how microglia react during the aging process, we profiled microglial transcriptomes via bulk RNA sequencing (RNA-seq) on fluorescence-activated cell sorting (FACS)-isolated microglia (CD11b^+^ CD45^low^) throughout the adult lifespan of C57BL/6J mice (3-, 6-, 9-, 12-, 14-, 16- and 24-month-old for females; 3-, 6-, 9-, 12-, 16-, 19- and 24-month-old for males) (Fig. 1a and Supplementary Fig. 1a). Biological replicates for RNA-seq were highly correlated in the same age (Supplementary Fig. 1b; *R* > 0.9 for both sexes, Pearson’s correlation). Bulk RNA-seq results were validated via quantitative polymerase chain reaction (qPCR) (Supplementary Fig. 1c). Our results revealed that the gene profiles of microglia from the female brain gradually changed during the aging process (Fig. 1b), indicating a progressive aging. Principal component analysis (PCA) further showed that female microglia can generally be divided into three groups: young (3-month-old), intermediate middle-aged (6-, 9-, 12- and 14-month-old) and aged (16- and 24-month-old) (Fig. 1d). Unexpectedly, microglia from male mice did not show a stepwise transition during the aging process or an intermediate middle-aged stage. Instead, male microglia displayed a young phenotype before 9-month-old. Thereafter, they precipitously switched to an aged phenotype after 12-month-old (Fig. 1c,e). Thus, female and male microglia exhibited different aging trajectories in the brain. Notably, we analyzed seven timepoints in each sex. Six out of seven timepoints are identical (3-, 6-, 9-, 12-, 16- and 24-month-old), and one timepoint in each sex is missing (19-month-old in female and 14-month-old in male). The missing timepoints are not in the stage transition. The potential influence from the discrepant timepoints is minimal in this study.

**Extended Data Fig. 1:**
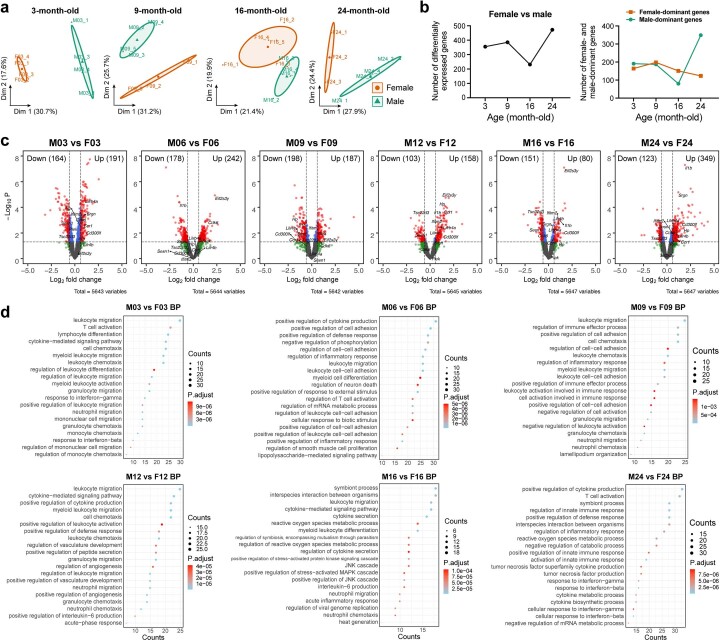
Sex differences in the microglial transcriptome at each age. **(a)** PCA plots display the sex differences in the microglial transcriptome at 3, 9, 16 and 24 months of age. **(b)** Numbers of differentially expressed genes at each age (left) and numbers of differentially expressed genes with higher expression in females or males (right). **(c)** Volcano plots of male vs. female microglial transcriptome changes at each age. Red points indicate DEGs. Log_2_FC ≥ Log_2_1.5, P < 0.05, quasi-likelihood (QL) F tests. **(d)** Top 20 enriched BP annotations by GO of the male vs. female microglial DEG profile at each age. Q < 0.05. Accumulative hypergeometric test with false discovery rate (FDR) correction. Dim, dimension. Source data

Previous studies revealed sex differences in microglial function, resulting in divergent transcriptome features and consequences in neurological disorders^49,50^. We next compared the transcriptomes between females and males at all matched ages (Extended Data Fig. 1a–c). At all examined ages, female and male microglia exhibited sex differences (Extended Data Fig. 1a–c). Hundreds of genes were differentially expressed between female and male microglia at each age (Extended Data Fig. 1b,c), 14 of which (*Il1b*, *Cd300lf*, *Ccr1*, *Lilr4b*, *Eif2s3y*, *Hp*, *Plek*, *Lilrb4a*, *Cdk6*, *Srgn*, *Samsn*, *Ifitm2*, *Tsc22d3* and *Sesn1*) were robustly differentially expressed between the two sexes across most ages (at least four out of six ages). Gene Ontology (GO) annotation further showed that the sexual divergence was mainly reflected in biological processes, including immune process regulation, cytokine-mediated signaling pathway, ossification, ROS metabolism and phagocytosis (Extended Data Fig. 1d). The sex differences may be partially attributed to the sex hormone regulation^49^.

Some differentially expressed genes (DEGs) were positively or negatively correlated with age, including the positively correlated *Axl* (female) and *Cd74* (male) and negatively correlated *Hspa13* (female) and *Ccl24* (male) in the mouse brain (Fig. 1f). We named them age-dependent microglia (ADEM) genes. We identified 57 and 14 ADEM genes positively correlated with age (P-ADEM genes) in female and male mice, respectively (Fig. 1g,h). P-ADEM genes include those participating in the interferon (IFN) signaling pathway (*Ifitm3*, *Ifi204*, *Cxcl16*, *Xaf1, Gas6* and *Tgtp2*), lipid metabolism (*Lpl*, *Apod* and *Spp1*), phagocytosis (*Axl*, *Spp1*, *Cst7* and *Fcgr3a*), defense responses (*Tnf*, *Il1rn*, *Ccl6* and *Ccl12*), ROS production (*Cybb* and *Hp*) and antigen presentation (*H2-D1*, *H2-Q7*, *Cd74*, *Tap1* and *H2-K1*) (Fig. 1g,h and Supplementary Table 1). We also identified 16 and 4 ADEM genes that were negatively correlated with age (N-ADEM genes) in female and male mice, respectively, including microglial marker genes (*Fcrls* and *Il4ra*), chemokine suppression and production genes (*Socs3*, *Tlr7* and *Il4ra*), endoplasmic-reticulum-associated protein degradation (ERAD)-associated genes (*Hspa13*) and iron transportation genes (*Fth1* and *Hfe*) (Fig. 1g-h and Supplementary Table 1). GO analysis revealed that the functions of ADEM genes were involved in leukocyte migration, chemokine production, IFN signaling, defense response, adaptive immune activation and innate immune activation (Fig. 1i and Supplementary Table 1). Ingenuity pathway analysis (IPA) further showed that the pathways related to LXR/RXR, FXR/RXR activation, α-tocopherol degradation, iron transport and iron homeostasis were enriched in ADEM genes (Fig. 1j and Supplementary Table 1), suggesting that aging may influence lipid and iron metabolism. Previous observations have indeed demonstrated that aging causes lipid and iron accumulation in microglia^51,52^. The functions of ADEM genes are consistent with DEGs found in aged mouse and human microglia^52–54^. Interestingly, most of male ADEM genes exhibited similar up-regulation and down-regulation trends with stepwise transitions in female microglia (Supplementary Fig. 2a), although the trends were not as prominent as the trends of female ADEM in female microglia (Fig. 1g). In contrast, most of female ADEM genes in male microglia presented a precipitous switch between young and aged stages (Supplementary Fig. 2b). The results double confirmed the gradual transition of female microglia and the precipitous switch of male microglia during the aging process.

To further decipher the characteristics of ADEM genes, we compared ADEM genes to the gene sets of disease-associated microglia (DAM)^55^ and activated response microglia (ARM)^56^. Among 85 ADEM genes, 21 genes overlapped with DAM genes (Fig. 1k). The fold changes (FCs) of overlapping genes in the ADEM and DAM subsets displayed a positive correlation (Fig. 1l). In contrast, only three ADEM genes (*Cd74*, *Cst7* and *Spp1*) were included in the ARM gene set (Fig. 1m), indicating that microglial aging is not simply equivalent to cell reactivity. The results indicate that ADEM genes are more likely to be linked to neurodegeneration, rather than microglial reactivity, suggesting active roles of microglia in neurological disorders of the aged brain.

### Characteristics of microglial cell aging in female mice at single-cell resolution

**Fig. 2:**
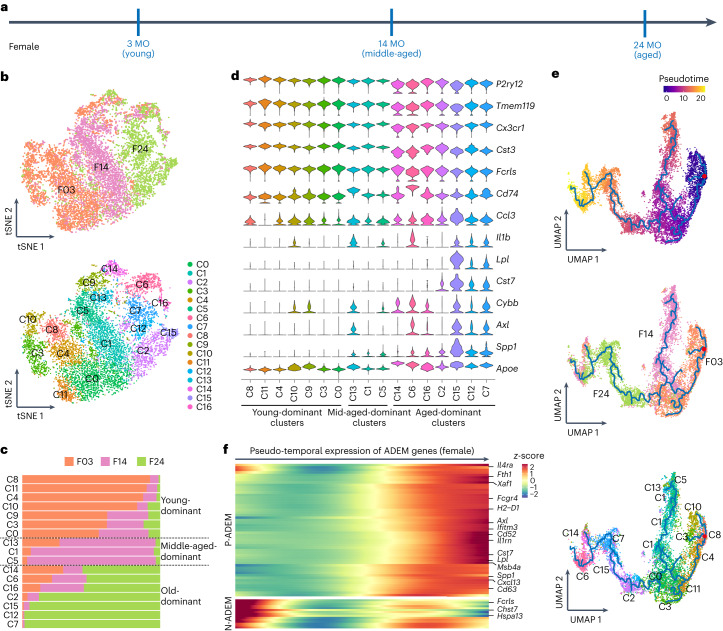
Characteristics of microglial cell aging by scRNA-seq. **a**, Scheme of microglial scRNA-seq from young (3 MO), middle-aged (14 MO) and aged (24 MO) female mice. **b**, tSNE plots of young (3 MO), middle-aged (14 MO) and aged (24 MO) microglia. Cells are divided into 17 clusters (C0–C16) by unsupervised classification, and cells are colored according to age (upper panel) and cluster (lower panel). **c**, Cell proportion of three ages in each cluster identifies young-dominant, middle-aged-dominant and aged-dominant clusters; clusters and age distributions are shown in **b**. **d**, Violin plots showing the expression levels of 14 microglial homeostasis-associated genes and inflammatory-related genes in each cluster. **e**, Pseudotime trajectories of microglia from three ages. Microglia at distinct time states show distinct trajectories. **f**, Heat map showing the expression levels of ADEM genes, which are correlated with pseudotime. *n* = 5 mice for each group. In total, 4,207 young (3 MO), 3,272 middled-aged (14 MO) and 2,497 aged (24 MO) microglia are harvested. F03, 3-month-old female, and so on in a similar fashion; MO, months old. Source data

To dissect how microglial cells age at single-cell resolution, we used 10x Genomics-based scRNA-seq to map the microglial transcriptome (Fig. 2a). Because only female microglia showed the stepwise aging process, whereas male microglia did not exhibit the progressive transition and intermediate middle-aged stage, the longitudinal analyses hereafter (including scRNA-seq and ATAC-seq) were primarily conducted in female mice to better display the aging process with the middle-aged microglia. We acquired 11,934 CD11b^+^ CD45^low^ cells from female brains in the young (3-month-old), middle-aged (14-month-old) and aged (24-month-old) stages with similar sequencing depth (Supplementary Fig. 3a). After the removal of low-quality reads, doublets and putatively dead cells (Supplementary Fig. 3b), cells were divided into five clouds (I–V) (Supplementary Fig. 3c). Based on brain cell markers, clouds I–V were annotated as microglia (87.02%), granulocytes (4.26%), T/natural killer (NK) cells (3.18%), macrophages/monocytes (5.14%) and astrocytes/oligodendrocytes (OLs) (0.40%) (Supplementary Fig. 3c,d). Thus, a total of 9,976 microglia from cloud I were obtained for subsequent analysis, including 4,207 young (3-month-old), 3,272 middled-aged (14-month-old) and 2,497 aged (24-month-old) microglia (Fig. 2b and Supplementary Fig. 3e). The scRNA-seq data were validated with bulk RNA-seq, immunostaining and RNAscope (Supplementary Figs. 4 and 5).

Based on unsupervised classification, microglia from three ages were divided into 17 clusters (C0–C16) (Fig. 2b and Supplementary Fig. 3e). Young microglia were predominantly located in C8, C11, C4, C10, C9, C3 and C0. Aged microglia were predominantly located in C14, C6, C16, C2, C15, C12 and C7. In contrast, microglia from middle-aged brains were mainly distributed in C13, C1 and C5 (Fig. 2b,c and Supplementary Figs. 3e and 7). The microglial homeostasis genes *P2ry12*, *Tmem119*, *Cx3cr1*, *Cst3* and *Fcrls* were expressed in all microglia, but they were highly enriched in young-dominant clusters (Fig. 2d). In contrast, *Cd74*, *Ccl3*, *Il1b*, *Lpl*, *Cst7*, *Cybb*, *Axl*, *Spp1* and *Apoe*, genes associated with neuroinflammation or diseases, were significantly up-regulated in the aged-dominant clusters (Fig. 2d). These results indicate that microglia undergo age-dependent alterations, switching from a homeostatic to a more inflammatory or diseased state during the aging process.

To precisely depict the development of ADEM genes during aging, we mapped signature scores of ADEM genes at single-cell resolution. Signature scores of both P-ADEM and N-ADEM genes were strongly correlated with age (Supplementary Fig. 7a,b). We further used pseudotime analysis to better visualize the transcriptional dynamics of the aging trajectory. Three age-dominant branches were identified, and C0, C1 and C2 were located at the key hub of the age dynamics transition (Fig. 2e). Notably, we found that the pseudo-temporal expression of most ADEM genes coincided with the up- or down-regulation trend (Fig. 2f), confirming that ADEM genes reliably represent core characteristics of microglial cell aging. Next, we projected ADEM-related GO biological functions on individual microglia, including the response to IFN, chemokine secretion and lipid localization (Supplementary Fig. 7c). ADEM genes associated with lipid metabolism were mainly enriched in aged microglia (Supplementary Fig. 7c), suggesting the dysfunction of lipid metabolism in the late lifespan. The transcriptional characteristics may explain previous observations of lipid metabolism deficits in aged microglia^28,57,58^.

### The chromatin landscape reveals the epigenetic regulation of ADEM genes

**Fig. 3:**
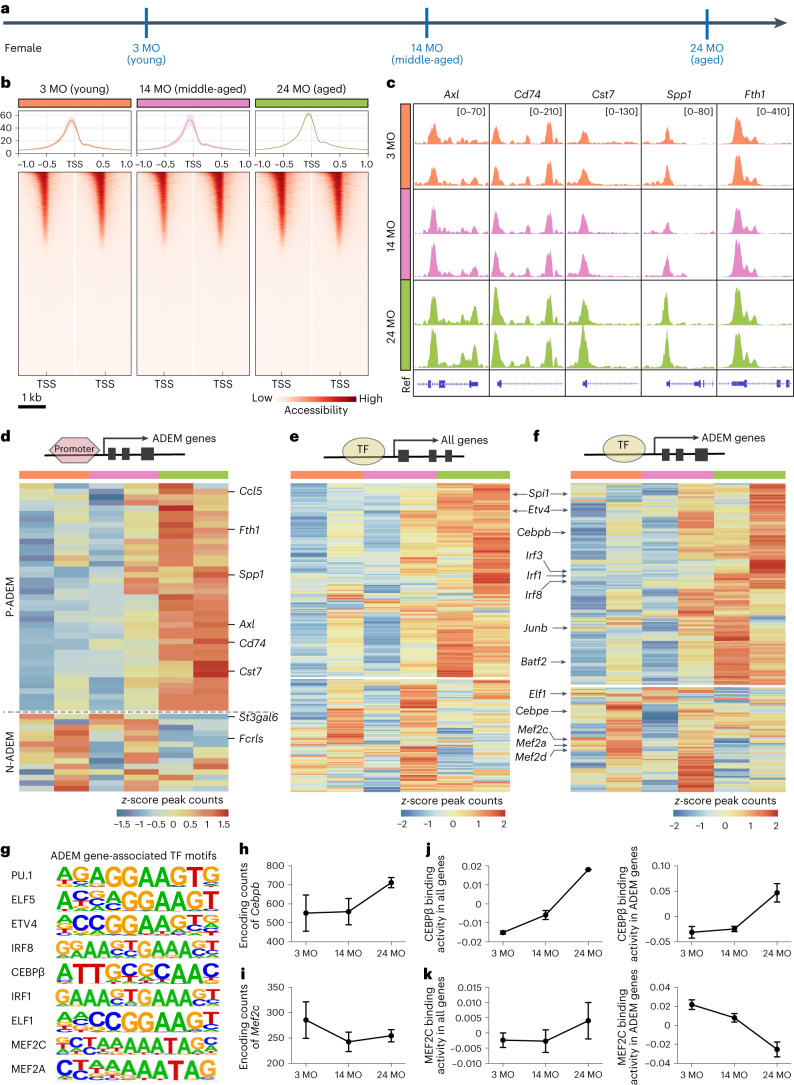
Chromatin landscapes identified by ATAC-seq unveil epigenetic features of microglia during the aging process. **a**, Scheme of microglial ATAC-seq from young (3 MO), middle-aged (14 MO) and aged (24 MO) mice. **b**, ATAC peaks around the TSS (−1 kb to 1 kb) of microglia at three ages. Data are presented as mean ± s.d., heat map showing enrichment of normalized ATAC-seq reads within ±1 kb of TSS in microglia at different stages. **c**, Representative genome browser views showing ATAC peaks of *Axl*, *Cd74*, *Cst7*, *Spp1* and *Fth1*. The numbers in brackets indicate the minimum and maximum values of the *y* axis. Ref, reference genome view of mm10. **d**, Heat map of differentially accessible chromatin promoter regions of ADEM genes. Cells are colored according to the *z*-score, and rows are hierarchically clustered. **e**,**f**, Heat map of differentially accessible TF motifs to all genes (**e**) and ADEM genes (**f**). Cells are colored according to the *z*-score, and rows are hierarchically clustered. **g**, Sequence logos of selected ADEM gene-associated TF motifs. **h**,**i**, Line chart showing the peak counts of the TF-encoding genes *Cebpb* (up-regulated with age) and *Mef2c* (down-regulated with age). **j**,**k**, Line chart showing the binding activities of CEBPβ (**j**) and MEF2C (**k**) to all genes and ADEM genes. Data are presented as mean ± s.d. *n* = 2 ATAC-seq tests for each group. Microglia from 2–3 mice were pooled together for each ATAC-seq test. MO, months old. Source data

**Extended Data Fig. 2:**
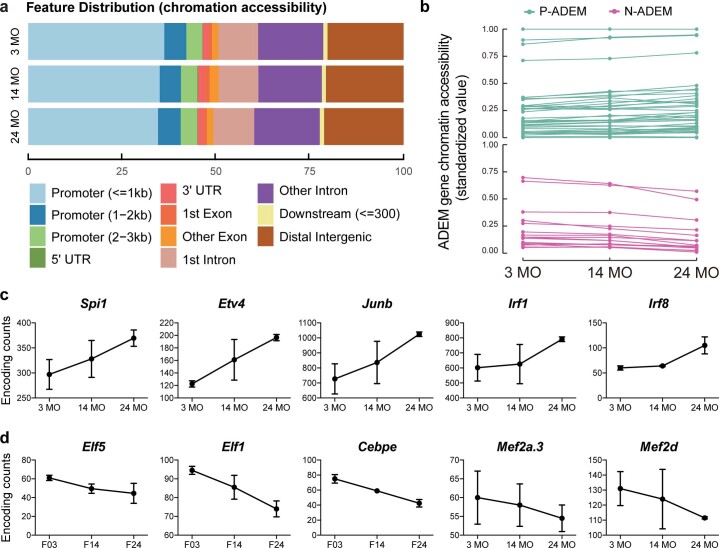
Chromatin modifications in the microglial aging process. **(a)** Percentages of differential chromatin accessibility in gene coding and regulatory elements at 3 ages. **(b)** Chromatin accessibility of ADEM genes at each age. Standardized values were calculated according to peak counts of the promoter region. **(c-d)** Encoding counts of genes encoding ADEM-enriched transcription factors at each age. Data are presented as mean ± SD. N = 2 ATAC-seq tests for each group. Microglia from 2 to 3 mice were pooled together for each ATAC-seq test. MO: month-old; ADEM: age-dependent microglia. Source data

Gene transcription is regulated by the chromatin landscape, including chromatin structure, DNA and histone modifications^59,60^. Accessible chromatin regions allow for the selective binding of regulatory elements, which is crucial to regulate gene expression in cell-type-specific or context-specific manners^60^. To explore potential mechanisms governing the transcriptional dynamics of microglia across the lifespan, we mapped the chromatin accessibility of female microglia in the young (3-month-old), middle-aged (14-month-old) and aged (24-month-old) stages (Fig. 3a) by ATAC-seq. ATAC peaks were aligned to gene regulatory regions throughout the whole genome. Peaks in microglia were mainly enriched ±1 kilobase (kb) around the transcription start site (TSS), primarily in the promoter region. The overall peak distribution in gene regulatory regions was similar among the three ages (Extended Data Fig. 2a). Previously, it was demonstrated that chromatin epigenetic features are relatively stable over time in microglia before the aged state^61^. In line with this, we observed a similar distribution of ATAC peaks within flanking regions identified in young and middle-aged microglia. Nonetheless, the number of ATAC peaks in aged microglia was increased (Fig. 3b), suggesting that microglia can respond to the aging state at the epigenetic level. Next, we asked whether ADEM gene expression is regulated by chromatin openness around promoter regions. To this end, we analyzed peak distributions in ADEM gene promoter regions. ADEM genes exhibited differential chromatin accessibility in different ages (Fig. 3c,d, Extended Data Fig. 2b and Supplementary Table 2). In general, the chromatin accessibility of P-ADEM genes increased when microglia became aged, whereas that of N-ADEM genes decreased (Extended Data Fig. 2b).

Transcription factors (TFs) are critical determinants of both transcriptional and epigenetic landscape alterations^62^. We annotated peaks to identify potential TFs accounting for the differential gene expression observed during the aging process. We first identified all differential chromatin binding sites of TFs and observed numerous TFs that displayed significantly higher activity in aged microglia (Fig. 3e). Next, we examined how TFs potentially govern the expression of ADEM genes during aging. We identified 50 TFs with significant enrichment in ADEM-accessible peaks across three ages, including key myeloid TFs (PU.1 and IRF8)^63^, immediate early response TFs (FOS, JUNB and cJUN)^64^, IFN pathway TFs (IRF1, IRF2 and IRF3)^65^, CEBP family members (CEBPβ and CEBPε)^66^ and ETS family members (MEF2A and MEF2C) (*P* < 0.05; Fig. 3f,g and Supplementary Table 2). Some of these TF peak counts were gradually up-regulated when microglia advanced in age, such as those of *Cebpb*, *Spi1* (encoding PU.1), *Etv4*, *Junb*, *Irf1* and *Irf8* (Fig. 3h and Extended Data Fig. 2c). In contrast, some of the peak counts were gradually down-regulated, including those of *Mef2c*, *Elf5*, *Elf1*, *Cebp3*, *Mef2a.3* and *Mef2d* (Fig. 3i and Extended Data Fig. 2d). We then assessed TF binding activities corresponding to ADEM peaks by using chromVAR^67^. Although the binding activities of both CEBPβ and MEF2C toward total genes displayed an age-dependent increasing trend, the binding activities of CEBPβ and MEF2C toward ADEM genes were enhanced and reduced, respectively (Fig. 3j,k). The results indicate that TFs selectively regulate gene expression in an orchestrated manner. A previous study showed that *Cebpb* was up-regulated in aged healthy brains and aged AD brains^68^. CEBPβ in microglia has been demonstrated to drive pro-inflammatory and neurodegeneration-related gene programs^66,69^. In contrast, MEF2C activity is implicated in enhancing cognitive function and conferring resilience to neurodegeneration^70^. The expression of MEF2C in microglia is down-regulated during brain aging in a type I interferon (IFN-1)-dependent manner. MEF2C limits the microglial inflammatory response to immune challenge. The loss of MEF2C results in, or is associated with, microglial priming^71^.

Taken together, our results indicate that chromatin accessibility and TF binding activity are responsible for regulating ADEM gene expression throughout the microglial lifespan. Accordingly, TF induction may act as a key regulator of the ADEM gene dynamics throughout the microglial lifespan.

### Aged microglia display a compromised molecular reaction to systemic LPS challenge

**Fig. 4:**
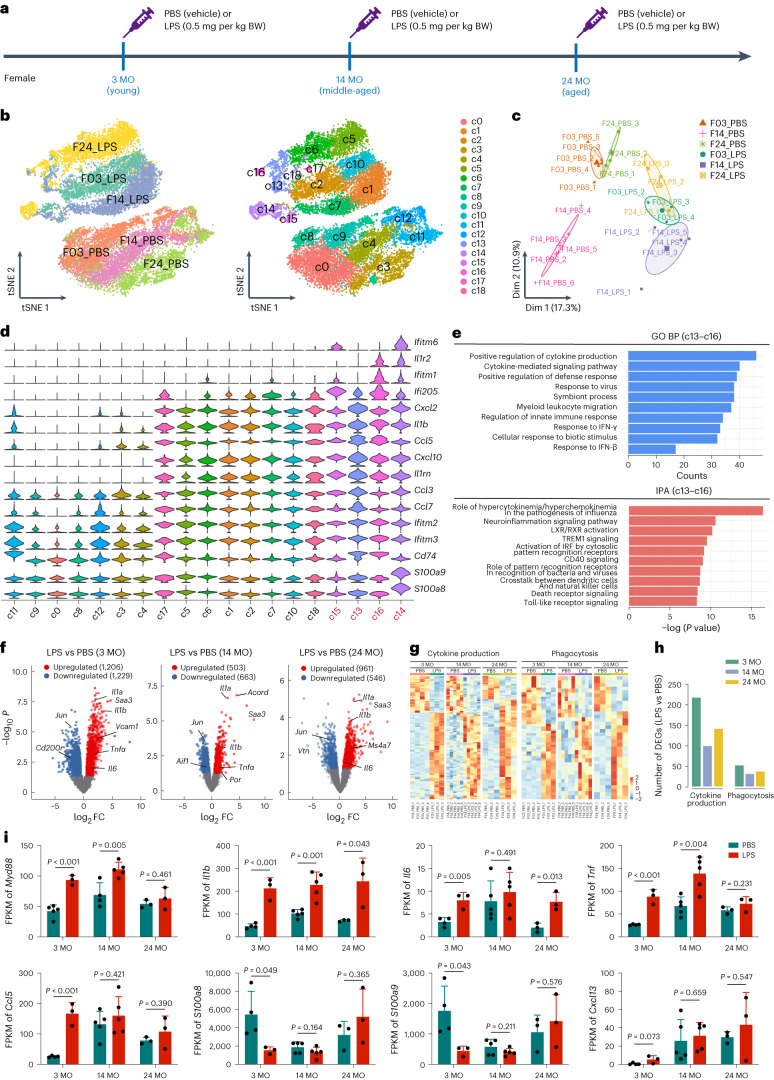
scRNA-seq and bulk RNA-seq reveal that microglia respond to LPS challenge in an age-dependent manner. **a**, Scheme of LPS and PBS administration and timepoints for scRNA-seq and bulk RNA-seq. **b**, tSNE plots of scRNA-seq display different responses of young (3 MO), middle-aged (14 MO) and aged (24 MO) microglia to LPS challenge. Cells are colored according to the group (left) and unsupervised clusters (right) separately. **c**, PCA plots of bulk RNA-seq display different responses of young, middle-aged and aged microglia to LPS challenge. *n* = 3–5 mice for each group. **d**, Violin plots show the expression levels of *Ifitm1*, *Ifitm6*, *Ilr2*, *Il1β*, *Ifitm2*, *Ccl5*, *Ccl7*, *Ccl3*, *Ccl4*, *Ccl12*, *Ifi205* and *Il1rn* grouped by clusters; these genes are enriched in c13–c16. **e**, Top 10 significant BPs annotated by GO and IPA of c13–c16 enriched genes (*q* < 0.05). **f**, Volcano plots of LPS-challenged microglia versus PBS-treated controls at 3 months, 14 months and 24 months of age. Red and blue represent significant DEGs (*P* < 0.05, log_2_FC ≥ 1, QL *F*-tests). **g**, Heat maps of cytokine production and phagocytosis-related DEGs of LPS-challenged microglia versus PBS-treated control at 3 months, 14 months and 24 months of age. **h**, Bar plot showing the DEG numbers of cytokine production-related and phagocytosis-related genes (as shown in **g**). **i**, FPKM of *Myd88*, *Il1b*, *Il6*, *Tnf*, *Ccl5*, *S100a8*, *S100a9* and *Cxcl13*. Data are presented as mean ± s.d. Two-tailed independent *t*-test. *n* = 5, 4, 3, 3, 5 and 3 mice for young (PBS), middle-aged (PBS), aged (PBS), young (LPS), middle-aged (LPS) and aged (LPS), respectively. Two-tailed independent *t*-test. *n* = 5 mice for each group of **b**, **d** and **e**. In total, 3,539 young (3 MO), 4,149 middle-aged (14 MO) and 4,313 aged (24 MO) microglia after LPS challenge were harvested in each group of **b**, **d** and **e**. BP: biological process; BW, body weight; F03_LPS, LPS-challenged 3-month-old females, and so on in a similar fashion; MO, months old; Dim, dimension. Source data

Previous work suggests that aged microglia are primed and respond in an exaggerated manner to a second stimulus^72–75^. However, whether the microglial transcriptomes of aged animals exhibit stronger alterations in response to inflammatory challenge remains unknown. To address this question, we treated young (3-month-old), middle-aged (14-month-old) and aged (24-month-old) female mice with LPS or PBS. Animals were euthanized 2 h after administration (Fig. 4a). scRNA-seq and bulk RNA-seq were used to investigate how microglia react to LPS challenge. Both scRNA-seq and bulk RNA-seq revealed that microglia of different ages responded differentially to LPS challenge. LPS-treated groups were primarily included in neighboring clouds rather than clustered with PBS-treated groups of the same age (Fig. 4b,c). Based on unsupervised classification, microglia from scRNA-seq data were divided into 19 clusters (c0–c18) (Fig. 4b). In the LPS-treated group (c1, c2, c5–c7, c10 and c13–c18), most clusters were composed of cells of the same age. Interestingly, c13–c16 consisted of cells of all three ages after LPS challenge (Fig. 4b). We found that IFN signaling activation-associated genes (*Ifitm1*, *Ifitm6* and *Ilr2*) were highly expressed in c13–c16 (Fig. 4d). Genes associated with IFN pathways (*Il1β*, *Ifitm2*, *Ccl5*, *Ccl7*, *Ccl3*, *Ccl4*, *Ccl12*, *Ifi205* and *Il1rn*) were enriched in c13–c16, indicating that these subsets of microglia were highly conserved across different ages and showed a strong response to LPS stimulation (Fig. 4d,e and Supplementary Table 3). Next, we asked whether ‘primed’ aged microglia display a stronger response to LPS challenge. We used bulk RNA-seq to compare LPS-treated and PBS-treated microglia at three ages (Fig. 4f). We identified 2,435 DEGs at 3 months of age, whereas the number reduced to 1,507 at 24 months of age (Fig. 4f and Supplementary Table 3). Upon LPS administration, 219 and 54 DEGs in young microglia were annotated to the cytokine production and phagocytosis categories, respectively (Fig. 4g,h and Supplementary Table 3). In contrast, only 143 and 39 genes in aged microglia were differentially expressed upon LPS challenge (Fig. 4g,h and Supplementary Table 3). The results indicate that aged microglia, which are considered primed, do not exhibit a more exaggerated response to LPS challenge. Similar results were also observed at the middle-aged stage (Fig. 4g,h). Therefore, aged and middle-aged microglia respond to LPS in a weaker/compromised manner.

**Extended Data Fig. 3:**
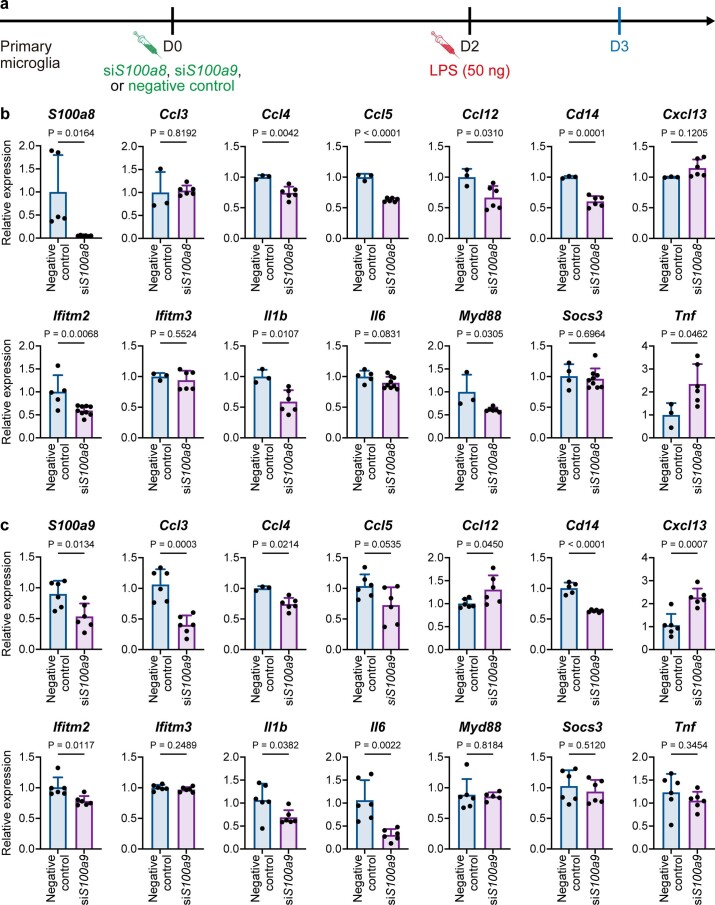
*S100a8* or *S100a9* knock-down down-regulates the LPS-induced immune response. **(a)** Scheme of Lipofectamine transfection and LPS challenge to primary microglia. **(b)** qPCR reveals that si*S100a8* transfection by Lipofectamine transfection successfully knocks down *S100a8*. Consequently, 7 out of 13 immune-related genes were down-reregulated upon LPS challenge. In the negative control group: *S100a8* (N = 5), *Ccl3* (N = 3), *Ccl4* (N = 3), *Ccl5* (N = 3), *Ccl12* (N = 3), *Cd14* (N = 3), *Cxcl13* (N = 3), *Ifitm2* (N = 5), *Ifitm3* (N = 3), *Il1b* (N = 3), *Il6* (N = 5), *Myd88* (N = 3), *Socs3* (N = 4) and *Tnf* (N = 3). In the si*S100a8* group: *S100a8* (N = 6), *Ccl3* (N = 6), *Ccl4* (N = 6), *Ccl5* (N = 6), *Ccl12* (N = 6), *Cd14* (N = 6), *Cxcl13* (N = 6), *Ifitm2* (N = 9), *Ifitm3* (N = 6), *Il1b* (N = 6), *Il6* (N = 9), *Myd88* (N = 6), *Socs3* (N = 9) and *Tnf* (N = 6). Where “N” corresponds to the number of biological replicates. **(c)** qPCR reveals that si*S100a9* transfection by Lipofectamine transfection successfully knocks down *S100a9*. Consequently, 8 out of 13 immune-related genes were down-reregulated upon LPS challenge. In the negative control group: *S100a9* (N = 6), *Ccl3* (N = 6), *Ccl4* (N = 6), *Ccl5* (N = 6), *Ccl12* (N = 6), *Cd14* (N = 5), *Cxcl13* (N = 6), *Ifitm2* (N = 6), *Ifitm3* (N = 6), *Il1b* (N = 6), *Il6* (N = 6), *Myd88* (N = 6), *Socs3* (N = 6) and *Tnf* (N = 6). In the si*S100a9* group: *S100a9* (N = 6), *Ccl3* (N = 6), *Ccl4* (N = 6), *Ccl5* (N = 6), *Ccl12* (N = 6), *Cd14* (N = 6), *Cxcl13* (N = 6), *Ifitm2* (N = 6), *Ifitm3* (N = 6), *Il1b* (N = 6), *Il6* (N = 6), *Myd88* (N = 5), *Socs3* (N = 6) and *Tnf* (N = 6). Where “N” corresponds to the number of biological replicates. Data are presented as mean ± SD. Two-tailed independent t test. Source data

Previous studies reported that LPS induces an exaggerated neuroinflammatory response in aged mice^72,76–80^. To further test whether aged microglia respond to LPS in an exaggerated manner, we evaluated the expression of several well-documented inflammation-related genes in microglia. We first analyzed *Myd88*, which controls the release of inflammatory factors. Although LPS significantly increased microglial *Myd88* levels at the young and middle-aged stages, it did not induce *Myd88* up-regulation at the aged stage (Fig. 4i). Next, we observed some key inflammatory mediators that are associated with neurotoxicity, including *Il1b* (ref. ^81^), *Il6* (ref. ^82^) and *Tnf*^83,84^. *Il1b* expression was significantly up-regulated after LPS challenge at all ages (Fig. 4i). However, LPS did not induce *Tnf* up-regulation in aged microglia (Fig. 4i). Notably, under homeostasis (PBS-treated groups), middle-aged microglia displayed higher *Il6* and *Tnf* levels than young microglia, whereas expression levels were reduced in aged microglia (Fig. 4i). Next, we analyzed the microglial expression of IFNγ response genes (*Ccl5*, *S100a8*, *S100a9* and *Cxcl13*), which were previously showed to display age-dependent up-regulation in brain tissue induced by an IL-1β, TNFα and IL12 mixture^74^. However, we failed to observe age-related up-regulation after LPS treatment (Fig. 4i). A previous study showed that S100A8 and S100A9 form the calprotectin heterocomplex, which is recruited by LPS-challenged TLR4 and subsequently induces deleterious pro-inflammatory cytokine release^85^. When we knocked down either *S100a8* or *S100a9* in primary microglia, the LPS-induced immune response was reduced, as seven of 13 and eight of 13 immune-related genes were significantly down-regulated upon *S100a8* and *S100a9* knock-down, respectively (Extended Data Fig. 3). The down-regulation of *S100a8* and *S100a9*, thus, can partially explained the weakened cytokine production observed in aged microglia. Therefore, despite their primed state, aged microglia exhibit a compromised immune response upon systemic challenge.

### Microglia show diverse alterations in their chromatin landscapes in response to aging and LPS challenge

**Fig. 5:**
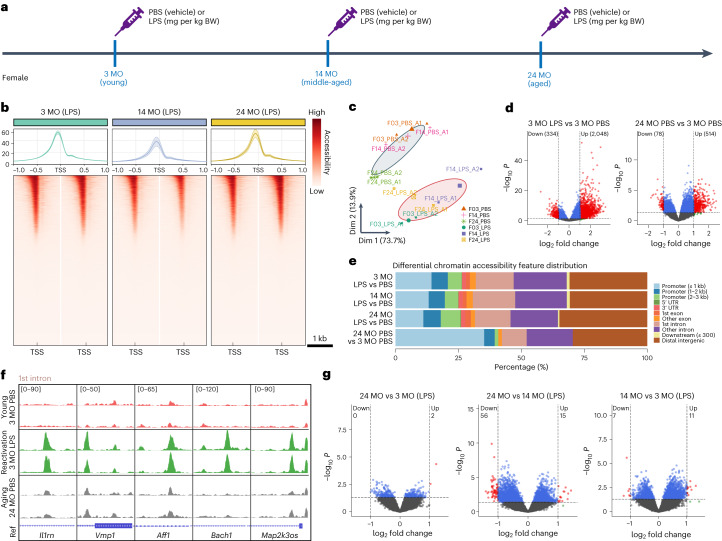
ATAC-seq reveals the divergence of chromatin modifications between microglial aging and reactivity to LPS challenge. **a**, Scheme of LPS and PBS administration and timepoints for ATAC-seq. **b**, ATAC peaks around the TSSs (−1 kb to 1 kb) of LPS-challenged microglia at three ages. Data are presented as mean ± s.d., heat map showing enrichment of normalized ATAC-seq reads within ±1 kb of TSSs in microglia at different stages. **c**, PCA plot showing that microglia of different ages exhibit distinct chromatin modifications in response to LPS challenge. **d**, Volcano plots of differentially accessible peaks upon LPS challenge (3 MO LPS versus 3 MO PBS) and age-related change (24 MO PBS versus 3 MO PBS), revealing divergent chromatin modifications between LPS challenge and the aging process (*P* < 0.05, log_2_FC ≥ 1, QL *F*-tests). **e**, Bar plots showing the distribution of differential peaks in gene encoding and regulatory element regions. **f**, Representative genome browser views showing ATAC peaks of *Il1rn*, *Vmp1*, *Aff1*, *Bach1* and *Map2k3os*. The numbers in brackets indicate the minimum and maximum values of the *y* axis. Ref, reference genome view, mm10. **g**, Volcano plots of differential accessible peaks of LPS-challenged microglia across different ages reveal conserved epigenetic modifications across different ages. *n* = 2 ATAC-seq tests for each group. Microglia from 2–3 mice were pooled together for each ATAC-seq test (*P* < 0.05, log_2_FC ≥ 1, QL *F*-tests). BW, body weight; MO, months old; UTR, untranslated region; Dim, dimension. Source data

Microglia undergo a mild and chronic reactive response in aged brains^86,87^, whereas LPS-induced reactivity is firm and transient^77,88^. To further decode differences between these two processes, we examined the epigenetic modulation in LPS-administered and PBS-administered microglia at young, middle-aged and aged stages (Fig. 5a,b). PCA results showed that both LPS challenge and the aging process induced substantial alterations in chromatin modifications, although the effect of LPS was more pronounced (Fig. 5c). In LPS-administered microglia, 44,353 peaks were identified and aligned to genomic loci. There were 2,382 differentially accessible peaks (DAPs) between LPS-administered and PBS-administered microglia at 3 months of age. In contrast, only 592 DAPs were identified between 24-month-old and 3-month-old microglia (Fig. 5d and Supplementary Table 4). Moreover, we examined the annotation of DAPs with ChIPseeker annotatePeak. DAPs found in LPS-treated versus PBS-treated microglia were primarily located in the first intron (Fig. 5e,f). In contrast, aging modulated DAPs, such that they predominately accumulated around promoter regions (Figs. 5e and 3c and Extended Data Fig. 2a). Interestingly, only a small number of chromatin accessibilities in LPS-challenged microglia were detected across different ages (Fig. 5g), indicating conserved epigenetic modifications upon LPS challenge across different ages. Therefore, these results indicate that aging and systemic inflammation induce distinct chromatin modulations.

### Microglial crosstalk with astrocytes and endothelial cells in the aged brain

**Extended Data Fig. 4:**
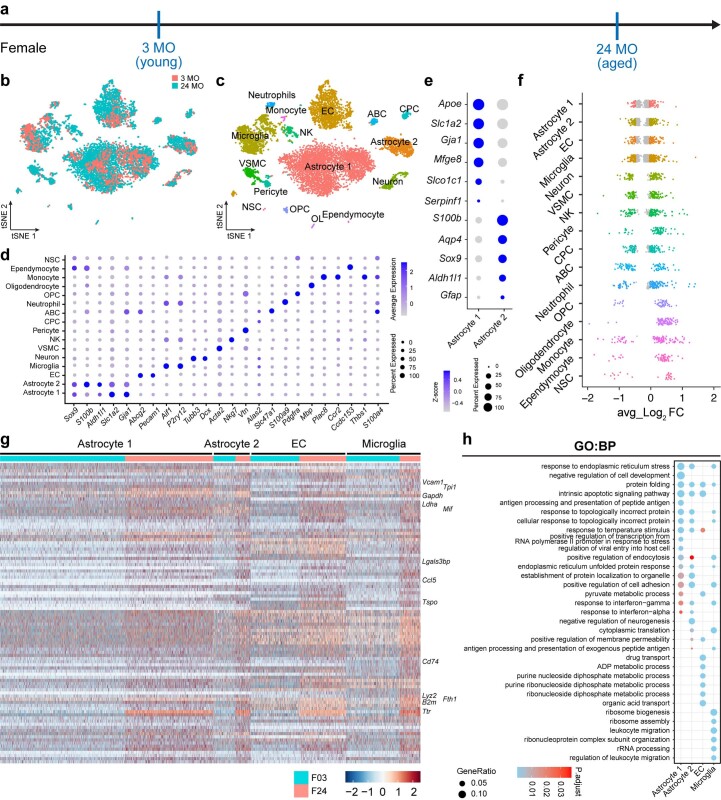
scRNA-seq characterizes the microglial cross talk with astrocytes and endothelial cells in the aged brain. **(a)** Scheme of brain scRNA-seq from young (3 MO) and aged (24 MO) mice. N = 5 mice for each group. **(b-c)** tSNE plots of 4,326 3 MO and 2,762 24 MO brain cells. Cells are colored according to age (b) and cell type (c) separately. **(d)** Dot plot showing the expression levels of well-known representative cell-type-enriched marker genes across all 15 cell types (16 populations) (see details in Methods). **(e)** Dot plot showing the expression levels of two astrocyte subtype marker genes. *Gja1*, *Mfge8*, *Apoe*, *Slc1a2* and *Slco1c1* were relatively enriched in Astrocyte 1, while *S100β*, *Sox9*, *Gfap* and *Aldh1l1* were highly expressed in Astrocyte 2. **(f)** Strip chart displaying 24 MO vs. 3 MO fold-changes in gene expression levels in each cell population. Colored dots indicate significantly up- or down-regulated (P < 0.05, log_2_FC ≥ 0.25) genes in aged cells compared to young cells. Non-parametric Wilcoxon rank sum test. **(g)** Heat maps of 24-month-old vs. 3-month-old DEGs (significantly up- or down-regulated (P < 0.05, log_2_FC ≥ 0.25) in no less than 2 populations) in Astrocyte 1 cells, Astrocyte 2 cells, EC and microglia. **(h)** Top significantly enriched biological processes of DEGs (significantly up- or down-regulated (P < 0.05, log_2_FC ≥ 0.25) annotated by GO (Q < 0.05) in Astrocyte 1, Astrocyte 2, EC and microglia. The accumulative hypergeometric test is used for statistical analysis with false discovery rate (FDR) correction. MO: month-old; GO: Gene Ontology; BP: biological process; EC: endothelial cell; VSMC: vascular smooth muscle cell; OL: oligodendrocyte; OPC: oligodendrocyte precursor cell; ABC: arachnoid barrier cell; NSC: neural stem cell; CPC: choroid plexus epithelial cell; NK: natural killer cell.

Different cell types in the brain cross talk and orchestrate^89^. The dysregulation of cell–cell communication is one of the hallmarks of aging^90^. As resident immune cells, microglia surveil and respond to microenvironmental alterations. However, how microglia affect their microenvironment and interact with other cells in the aged brain are not fully understood. To better understand how aging impacts on microglia and other brain cells, we predicted the intercellular signaling and cell–cell communication. Brain cells from young (3-month-old) and aged (24-month-old) mice were harvested for scRNA-seq (Extended Data Fig. 4a). After removing low-quality cells and red blood cells, we finally acquired 9,088 cells for subsequent analysis (Extended Data Fig. 4b and Supplementary Fig. 8). We identified 15 distinct cell populations according to cell-type-specific gene signatures^42^ (Extended Data Fig. 4c,d and Supplementary Fig. 8). Two astrocyte populations were identified (Extended Data Fig. 4c,g and Supplementary Fig. 8). *Apoe*, *Slc1a2, Gja1*, *Mfge8* and *Slco1c1* were highly expressed in Astrocyte 1, whereas *S100β*, *Aqp4*, *Sox9*, *Aldh1l1* and *Gfap* were relatively enriched in Astrocyte 2 (Extended Data Fig. 4e), consistent with previous observations^91^. Notably, the major cell types collected by our method were microglia, astrocytes and endothelial cells (ECs) (Extended Data Fig. 4c,f and Supplementary Fig. 8a,b); therefore, we investigated the overlapping DEGs in these cell types. *Ttr*, *Crip1*, *Ly6a*, *Mgp*, *B2m*, *Tspo* and *Vcam1* were up-regulated in most cell types (Extended Data Fig. 4g and Supplementary Table 5). *Ttr* was prominently up-regulated in aged brains. TTR is a transporter of vitamin A and thyroxine. A previous study demonstrated that TTR is neuroprotective after brain injury^92^. On the other hand, TTR is also a risk factor for the formation of amyloid fibrils in familial amyloid polyneuropathy^93^, implying that the high expression of *Ttr* contributes to neurodegeneration in the aged brain. In addition, we identified the most common biological functions enriched in each cell population, which include the ‘response to interferon signaling’, ‘protein synthesis’, ‘response to stress’ and ‘regulation of cell apoptosis and cell cycle’ (Extended Data Fig. 4h and Supplementary Table 5).

Previous studies showed aging-driven deterioration of brain structures and functions, particularly in the BBB^94,95^. Dysfunction or breakdown of the BBB results in the entry of neurotoxic factors, leading to neuroinflammation^96^. Thus, we highlighted the cellular interactions between microglia and BBB components, including astrocytes and ECs. The gene profiles of astrocytes and ECs underwent evident alterations in the aged brain (Extended Data Fig. 4f,g and Supplementary Fig. 9). Genes associated with antigen presentation (*B2m*, *Cd74* and *Hla-a*), oxidative stress (*Alpl*, *Fos*, *Hsp90ab1* and *Jun*), glycolysis and hypoxia response (*Aldoa*, *Pkm*, *Tpl1*, *Edn1* and *Ldha*) and neuroinflammation signaling (*Vcam1*, *cd200* and *Vmf*) were up-regulated in aged ECs (Supplementary Fig. 9a and Supplementary Table 6), indicating the involvement of ECs in the overall inflammatory state during brain aging. In aged astrocytes, the levels of major histocompatibility complex (MHC) class I/II genes (*B2m*, *H2-D1*, *Cd74* and *MHCII* genes involved in adaptive immunoregulation) and the complement cascade gene *C4b* were markedly elevated (Supplementary Fig. 9b and Supplementary Table 6). However, *Occludin*, *Claudins* and *Cx4*, which encode key BBB component proteins^97,98^, did not show significant alterations during the aging process (Supplementary Table 6). Thus, BBB dysfunction might not be driven by intrinsic cellular changes but, rather, by the inflammatory microenvironment or the recruitment of immune cells to the gliovascular unit, which increase susceptibility to age-related disorders.

**Extended Data Fig. 5:**
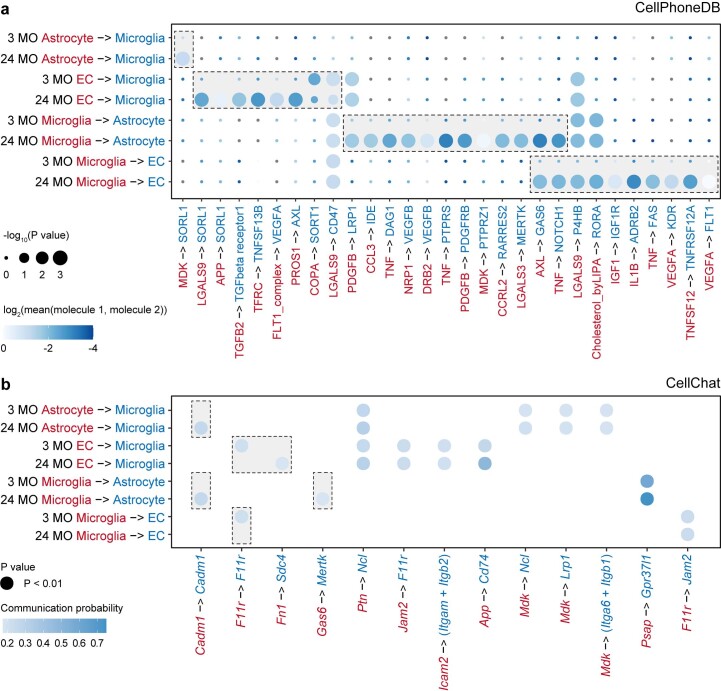
Predicted molecule-molecule interactions among microglia, astrocytes and ECs in young and aged mice. **(a)** Age-dependent ligand-receptor interactions predicted by CellPhoneDB. One-sided permutation test. **(b)** Significant age-dependent molecule-molecule interactions predicted by CellChat. MO: month-old; EC: endothelial cell. One-sided permutation test.

To further compare the altered cell signaling during the aging process, we additionally applied CellPhoneDB and CellChat^99–101^ to predict the cell–cell interaction. By CellPhoneDB, we identified 29 age-dependent ligand–receptor interactions (defined as in a specific cell–cell interaction, *P* < 0.01 in a certain age, and *P* > 0.01 in the other age) (Extended Data Fig. 5a). TNF, TGF-β, PDGF and VEGF signaling pathways were differentially involved in microglia during the aging process (Extended Data Fig. 5a). In addition, we identified 13 significant transmitter–receiver interactions by CellChat, four of which were differentially involved between different ages (Extended Data Fig. 5b).

### Forced microglial turnover induced by 3xDR accelerates microglial cell aging

**Fig. 6:**
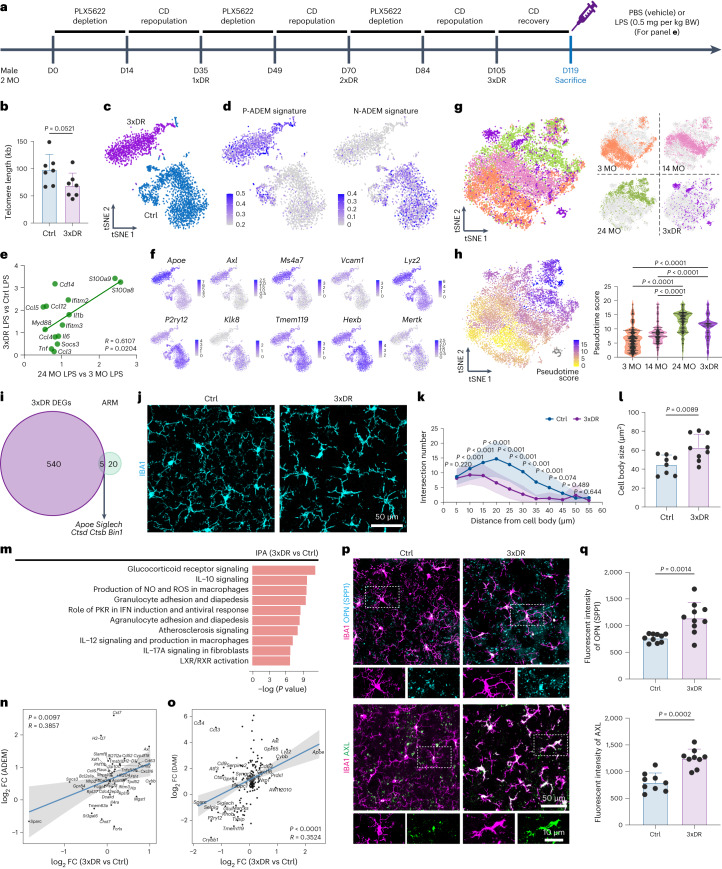
3xDR microglia exhibit an aged-like phenotype. **a**, Scheme of 3xDR and timepoints for experiments. **b**, 3xDR shortens the microglial telomere. *n* = 7 mice for each group. Two-tailed independent *t-*test. **c**, 3xDR microglia (1,313 cells) exhibited transcriptional characteristics distinct from control mice (2,180 cells). *n* = 5 mice for each group. **d**, 3xDR microglia display higher P-ADEM and lower N-ADEM gene signature scores. **e**, 3xDR versus control microglia FC (qPCR) exhibited a similar trend to LPS challenge as aged versus young microglia FC (bulk RNA-seq). *n* = 4 (3xDR and control) and 3 (3-month-old and 24-month-old; data from Fig. 4) mice. Linear regression. **f**, tSNE plots show the expression levels of microglial activation-associated and homeostasis-associated genes. **g**, 3xDR microglia exhibit a phenotype resembling that of aged microglia. **h**, 3xDR and 24-month-old microglia exhibit higher pseudotime value than 3-month-old and 14-month-old microglia. One-way ANOVA with Bonferroni’s post hoc test. *n* = 5 mice for each group. In total, 4,207 young, 3,272 middled-aged, 2,497 aged and 1,313 3xDR microglia (**g**,**h**). **i**, Only five genes are overlapping between 3xDR DEGs and ARM gene set. **j**–**l**, 3xDR microglia exhibit a distinct morphology to control microglia. 3xDR microglia display a larger cell body than control microglia. *n* = 8 mice for control and 9 mice for 3xDR, 100 cells from cortex and hippocampus for each group. Each dot represents an average result in one mouse (**l**). Two-tailed independent *t*-test. **m**, Top 10 significantly enriched canonical pathways of 3xDR versus control microglial DEGs annotated by IPA (q < 0.05). **n**,**o**, 3xDR versus control microglia FC are positively correlated with ADEM (**n**) and DAM (**o**) gene sets. Gray shading represents the 95% confidence interval, and Pearson’s correlation coefficients and *P* values are shown at the bottom. **p**,**q**, Representative confocal image shows that OPN and AXL are up-regulated in 3xDR microglia. *n* = 10 mice for each group. Two-tailed independent *t*-test. Data are presented as mean ± s.d. BW, body weight; Ctrl, control; MO, months old; NxDR, N-round depletion–repopulation; PLX5622, PLX5622-formulated diet. Source data

**Extended Data Fig. 6:**
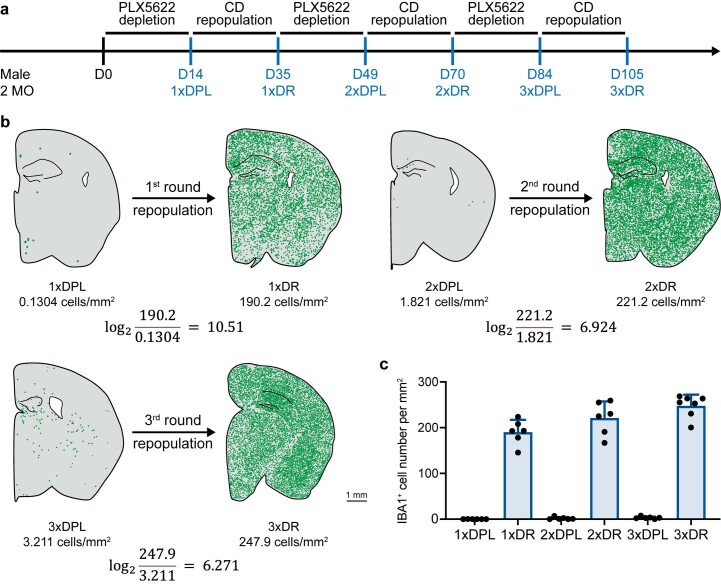
Each microglial cell proliferates several times by 3xDR. **(a)** Scheme of the forced microglial turnover model and timepoints for experiments. **(b)** Representative spatial distribution of microglia in 3 rounds of depletion-repopulation. Each green dot represents an IBA1^+^ microglial cell. **(c)** Statistics of IBA1^+^ cell number in each round of microglial depletion-repopulation. Data are represented as the mean ± SD. N = 6 (1xDPL, 2xDPL, 3xDPL, 1xDR and 2xDR) to 7 mice (3xDR) for each group. PLX5622: PLX5622-formulated diet; CD: control diet; NxDPL: N-round depletion; NxDR: N-round depletion-repopulation. Source data

**Extended Data Fig. 7:**
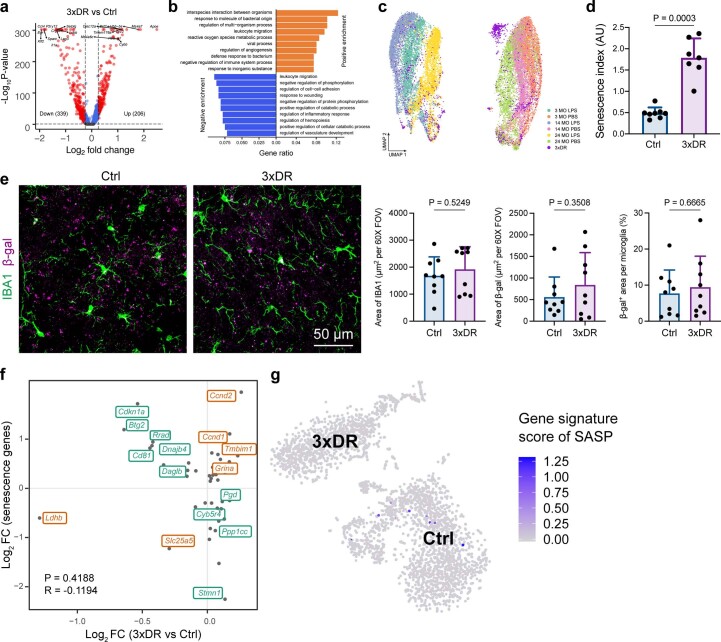
Despite the increased morphology-based senescence index, 3xDR microglia do not display a senescent state at the transcriptional level. **(a)** Volcano plots of DEGs in 3xDR vs. control microglial transcriptomes (log_2_ FC > 0.25, adjusted P value < 0.05). Non-parametric Wilcoxon rank sum test. **(b)** Top 10 significantly enriched biological processes (Q < 0.05) in 3xDR vs. control microglia. The orange bar represents up-regulated gene-enriched pathways; the blue bar represents down-regulated gene-enriched pathways. **(c)** UMAP plot of LPS-treated, PBS-treated and 3xDR microglia, revealing that 3xDR microglia do not exhibit a reactive phenotype. **(d)** Senescence index of microglia in the control and forced turnover groups, calculated as the area of IBA1 (mm^2^) divided by the area under the curve (AUC). Data are represented as the mean ± SD. N = 8 and 7 mice for control and 3xDR, respectively. Two-tailed independent t test. **(e)** Quantification of β-gal levels in cortical and hippocampal microglia. IBA1 area, β-gal area or β-gal^+^ microglia are not changed in 3xDR mice. Data are presented as mean ± SD. N = 9 mice for each group, 60 cells in total. Two-tailed independent t test. **(f)** Scatter plot showing the correlation between well-known senescence genes and the 3xDR microglial phenotype, indicating that well-defined senescence genes are not correlated with 3xDR microglia. Pearson correlation. **(g)** 3xDR and control microglia exhibit similar SASP gene signature scores. PLX5622: PLX5622-formulated diet; CD: control diet; Ctrl: control; 3xDR: 3-round depletion-repopulation; FC: fold change; SASP: senescence-associated secretory phenotype; FOV: field of view; AU: arbitrary unit. Source data

**Extended Data Fig. 8:**
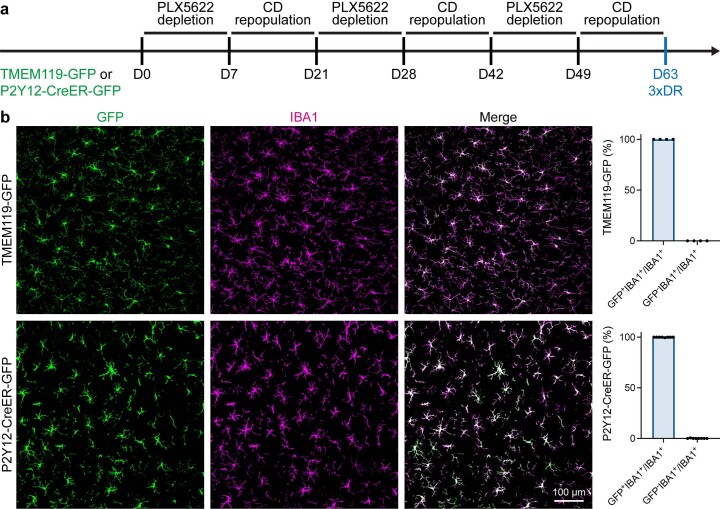
3xDR IBA1^+^ brain parenchymal cells are microglia instead of BAMs or infiltrating myeloid cells. **(a)** Scheme of the 3xDR forced microglial turnover model. **(b)** Almost all IBA1^+^ cells are GFP^+^ microglia in 3xDR TMEM119-GFP and P2Y12-CreER-GFP mice. N = 4 mice for TMEM119-GFP and N = 8 for P2Y12-CreER-GFP mice. Data are presented as mean ± SD. PLX5622: PLX5622-formulated diet; CD: control diet; NxDR: N-round depletion-repopulation. Source data

We administered male mice a PLX5622-formulated diet for 14 d to deplete brain microglia, followed by control diet (CD) for 21 d for repopulation (Extended Data Fig. 6a). After three rounds of depletion–repopulation, each microglia proliferated at least log_2_(190.2/0.1304) + log_2_(221.2/1.821) + log_2_(247.9/3.211) = 23.71 times on average (Extended Data Fig. 6b,c). To ensure that microglia reached a steady state, we extended the analysis for another 14 d after the third round of depletion–repopulation to allow microglia to fully recover (Fig. 6a). Aging leads to telomere shortening^46,47^. The telomere length of 3xDR microglia displayed a similar shortened trend (Fig. 6b). 1xDR microglia (aka repopulated microglia) display similar morphology, transcriptome profile and function to naive microglia^41,45,102–104^. In contrast, gene characteristics of 3xDR microglia were distinct from CD-treated control (Fig. 6c). 3xDR microglia exhibited higher P-ADEM and lower N-ADEM gene signature scores (Fig. 6d). In line with this, 3xDR microglia exhibited a similar trend upon LPS challenge as LPS-challenged aged microglia (Fig. 6e), indicating an aged-like phenotype. A total of 545 genes were differentially expressed, among which 206 were up-regulated, including genes involved in DAM (*Apoe*, *Axl*, *Spp1*, *Ms4a7* and *Vcam1*), immune activation (*Lyz2*, *Tspo*, *Il1b*, *Il10*, *Cst7*, *Ifi203*, *Ifitm3* and *Ifitm6*) and ROS production (*Gsr* and *Prdx5*). In contrast, 339 genes were down-regulated, such as genes involved in microglial homeostasis (*P2ry12*, *Klk8*, *Tmem119*, *Hexb, Sall1* and *Cx3cr1*) and phagocytosis/phagosome maturation (*Mertk* and *Ctsf*) (Fig. 6f, Extended Data Fig. 7a,b and Supplementary Table 7). The results indicate that 3xDR microglia in the 6-month-old brain lose their homeostatic state and exhibit a dampened phagocytic capacity. We further replotted the male 3xDR microglia with cells from young, middle-aged and aged female mice. 3xDR microglia were mainly located in the proximal cloud of aged microglia and exhibit a higher pseudotime score than young and middle-aged microglia (Fig. 6g,h). When plotted with LPS-treated and PBS-treated microglia, 3xDR microglia primarily located close to PBS-treated microglia (Extended Data Fig. 7c). In addition, only five 3xDR DEGs were included in the ARM gene set (Fig. 6i). These results confirmed that 3xDR microglia do not exhibit an LPS-induced reactive state. On the other hand, 3xDR microglia exhibited a less ramified morphology with less complicated processes and enlarged cell bodies (Fig. 6j,l), indicating an aged-like state^105^. Several enriched pathways were identified by IPA, including glucocorticoid receptor signaling, IL-10 signaling, IL12 and IL17a signaling, NO and ROS production, PKR in IFN induction, LXR and RXR activation and the NRF2-mediated oxidative stress response (Fig. 6m and Supplementary Table 7). Next, we compared the DEGs of 3xDR microglia to ADEM genes and found a positive correlation (Fig. 6n), echoing the aged-like phenotype of 3xDR microglia. Similar to aged microglia, we identified a positive correlation between 3xDR microglia and DAM genes (Fig. 6o), suggesting potential associations with brain disorders. The aged-like state of 3xDR microglia was further confirmed by the immunostaining of OPN (encoded by *Spp1*) and AXL (Fig. 6p,q). Notably, 44 metabolism-related biological processes were enriched in 3xDR microglial DEGs (Supplementary Fig. 10), suggesting that the metabolism status of microglia was influenced by 3xDR. To confirm whether the repopulated cells in 3xDR are microglia, we used microglia-specific mice, TMEM119-GFP and P2Y12-CreER-GFP (Extended Data Fig. 8a), in which only microglia expressed GFP reporter, whereas border-associated macrophages (BAMs) or infiltrating myeloid cells do not^41,106,107^. In both 3xDR TMEM119-GFP and P2Y12-CreER-GFP animals, almost all IBA1^+^ cells in the brain parenchyma were GFP^+^ (Extended Data Fig. 8b), confirming the microglial identity of repopulated cells in the 3xDR brain. Notably, some comparisons were between male 3xDR and female young/mid-aged/aged microglia (Fig. 6g,h and Extended Data Fig. 7c). To further confirm the aged-like phenotype of male 3xDR microglia, we compared some age-related scores from bulk RNA-seq data between female and male microglia. Female and male microglia at both young and aged stages showed similar ADEM signature and pseudotime scores (Supplementary Fig. 11). Albeit we compared the pseudotime score and replotted the *t*-distributed stochastic neighbor embedding (tSNE) between male 3xDR and female young/mid-aged/aged microglia, the similar age-related scores between sexes infer the aged-like characteristics of male 3xDR microglia. Collectively, forced proliferation by 3xDR switches microglia to an aged-like phenotype, even though the microglia are in a non-aged brain.

We next asked whether aged-like 3xDR microglia exhibit a senescent phenotype. Molecular markers for microglial senescence are currently not well defined. A morphology-based senescence index was used to evaluate the microglial state, by which aged microglia exhibit a higher senescence index^105^. The senescence index of 3xDR microglia was 3.6-fold that of normal microglia (Extended Data Fig. 7d). Additionally, we compared cytoplasmic β-galactosidase (β-gal), a widely used marker of cell senescence, between control and 3xDR microglia. However, we did not observe β-gal elevation in 3xDR microglia (Extended Data Fig. 7e). The conflicting results, although the ‘senescence index’ does not technically distinguish senescence and aging, thus complicate the landscape of the microglial senescence state. To fully elucidate whether aged-like 3xDR microglia exhibit a senescent phenotype, we compared a series of widely accepted senescence genes^48,108,109,110,111^. We found that these senescence genes were not correlated with DEGs of 3xDR microglia (Extended Data Fig. 7f). In addition, 3xDR microglia did not exhibit a different gene signature score of senescence-associated secretory phenotype (SASP)^112,113^ from control microglia (Extended Data Fig. 7g). Converging results thus indicate that aged-like 3xDR microglia did not exhibit a senescent phenotype.

Consequently, forced turnover by 3xDR converts microglia into an aged-like but non-senescent state.

### Accelerated microglial cell aging dampens cognitive functions

Microglia are essential for cognitive functions^114,115^, and aging dampens cognitive functions. However, the contribution of aged microglia to cognitive declines remains elusive, partially because of difficulties in dissecting the contribution of aged microglia from other aged brain cells. Here, we established an accelerated microglial aging model by 3xDR. Because CSF1R is specifically expressed in microglia and macrophages, other brain cells are not directly influenced by 3xDR. The aged-like 3xDR microglia thus reside in a non-aged microenvironment, allowing to dissect the contribution of aged-like microglia per se without the influence of aged microenvironment. We thus used 3xDR to investigate the contribution of aged-like microglia to cognitive functions.

**Fig. 7:**
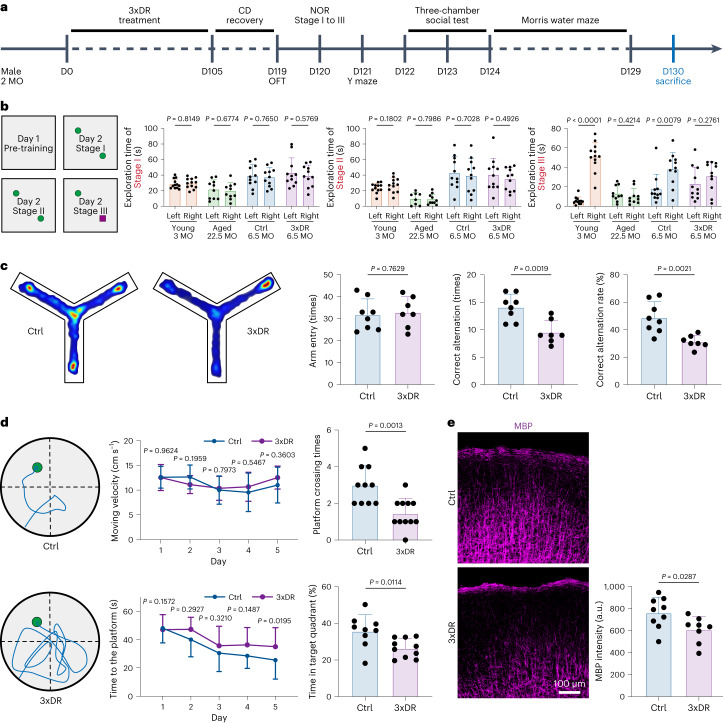
3xDR leads to cognitive decline and myelination impairment in non-aged mice. **a**, Scheme of the 3xDR mice preparation and behavior tests. **b**, NOR reveals that 3xDR induces cognitive decline in recognition memory, resembling the phenotype of aged mice. Left, the paradigm of NOR; right, the mouse exploration time to two objects. *n* = 12, 10, 11 and 11 mice for young, aged, control and 3xDR groups, respectively. **c**, Y maze reveals that 3xDR induces cognitive decline in spatial learning. Left, representative trajectory heat map in the Y maze; middle and right, the statistical results of control and 3xDR mice. *n* = 10 mice for each group. **d**, Morris water maze reveals that 3xDR induces cognitive decline in spatial learning. Left, representative swimming route of control and 3xDR mice in the Morris water maze; right, latency in the training phase, the number of times the mice passed across the platform and the time in the target quadrant in the probe trial. *n* = 10 and 11 mice for control and 3xDR, respectively. **e**, Representative confocal images of MBP in the cortex and quantification of average MBP expression in control and 3xDR mouse brains. 3xDR impairs myelination in the cortex. *n* = 9 and 8 mice for control and 3xDR, respectively. Data are presented as mean ± s.d. Two-tailed independent *t*-test. Ctrl, control; OFT, open field test. Source data

**Extended Data Fig. 9:**
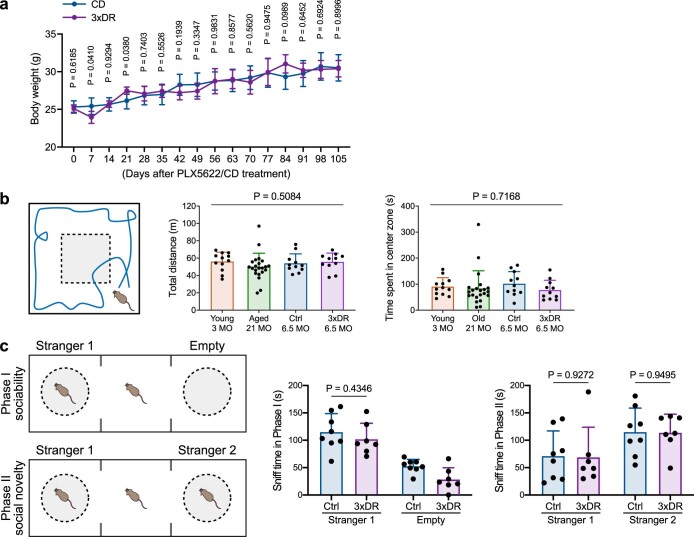
3xDR does not increase anxiety level or social deficit. **(a)** 3xDR does not influence the body weight of mice. N = 5 mice for each group. Multiple t test. **(b)** Open field test reveals that the total distance and time spent in the center zone were comparable among young (3-month-old), aged (21-month-old), control and 3xDR mice, indicating that 3xDR does not increase anxiety-like behavior. N = 12, 22, 11 and 11 for young, aged, control and 3xDR mice, respectively. One-way ANOVA. **(c)** Three-chamber test shows that sniff times in the sociability stage and social novelty stage are not changed in 3xDR mice, indicating that 3xDR does not exhibit the social deficit. N = 8 for control mice and 7 for 3xDR mice. Two-tailed independent t test. Data are presented as mean ± SD. Ctrl: control; 3xDR: 3-round depletion-repopulation. Source data

The 3xDR procedure did not affect the body weight of the mice (Extended Data Fig. 9a). The open field test revealed that the general motor ability and anxiety level were also not influenced by 3xDR (Extended Data Fig. 9b). In addition, the social preference test showed that 3xDR-treated mice did not exhibit social deficits (Extended Data Fig. 9c). We then asked whether 3xDR influences learning and memory (Fig. 7a). To this end, we first performed the novel object recognition (NOR) to test the recognition memory of young (3-month-old), aged (22.5-month-old), 3xDR and age-matched control mice. Young and control mice spent more time on exploring the novel object in Stage III. In contrast, both aged and 3xDR mice did not preferentially explore the novel object (Fig. 7b). Second, we used the Y maze to assess the spatial working memory. Although the arm entry times were unchanged, both correct alternation and the alternation rate decreased after 3xDR treatment (Fig. 7c), indicating deficits in spatial working memory. Third, we used the Morris water maze to evaluate spatial long-term memory. Although the moving velocity was unchanged, 3xDR mice spent more time on finding the platform in the training phase and less time in the target quadrant in the probe test phase (Fig. 7d). Results from the NOR, Y maze and Morris water maze tests demonstrate that 3xDR leads to cognitive declines in learning and memory. Therefore, accelerated microglial aging mediated by forced turnover directly dampens learning and memory, indicating the vital role of microglia in cognitive functions.

**Extended Data Fig. 10:**
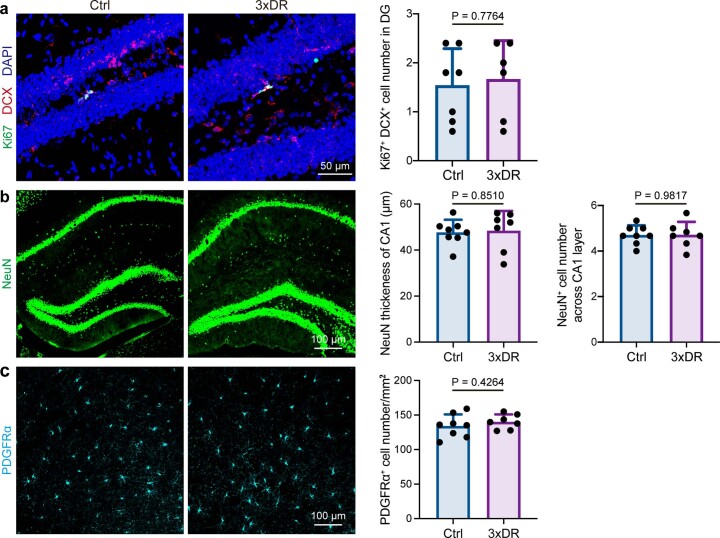
3xDR does not influence the adult neurogenesis, neuron number or OPC density. **(a)** Quantification of Ki67^+^ DCX^+^ cell numbers in microglia in the dentate gyrus. 3xDR does not influence adult neurogenesis in the dentate gyrus. N = 7 and 6 mice for control and 3xDR, respectively. **(b)** NeuN quantification in CA1. 3xDR does not influence the NeuN thickness of CA1. N = 8 and 7 mice for control and 3xDR, respectively. **(c)** 3xDR does not influence the OPC density. N = 8 and 7 mice for control and 3xDR, respectively. Data are presented as mean ± SD. Two-tailed independent t test. Ctrl: control; 3xDR: 3-round depletion-repopulation. Source data

Next, we investigated the potential mechanism by which the aging of 3xDR microglia dampens learning and memory. We examined neurogenesis in the hippocampus and did not identify a significant difference of Ki67^+^ DCX^+^ cells in the dentate gyrus or mature neurons in CA1 (Extended Data Fig. 10a,b). Recent studies demonstrated that myelination in the adult brain contributes to spatial memory^30,116^. Hence, we examined myelin basic protein (MBP) in the brain cortex. In layers I–III, the MBP intensity was significantly reduced in 3xDR mice (Fig. 7e). In contrast, the number of oligodendrocyte precursor cells (OPCs) was not changed in the cortex (Extended Data Fig. 10c). Microglia are capable of modulating OPC/OL differentiation^117,118^. Therefore, our results indicate that aged microglia may lose the capacity to facilitate OPC/OL differentiation. The reduction of myelination in turn impairs learning and memory.

### 3xDR-induced microglial aging influences the cell–cell interaction

**Fig. 8:**
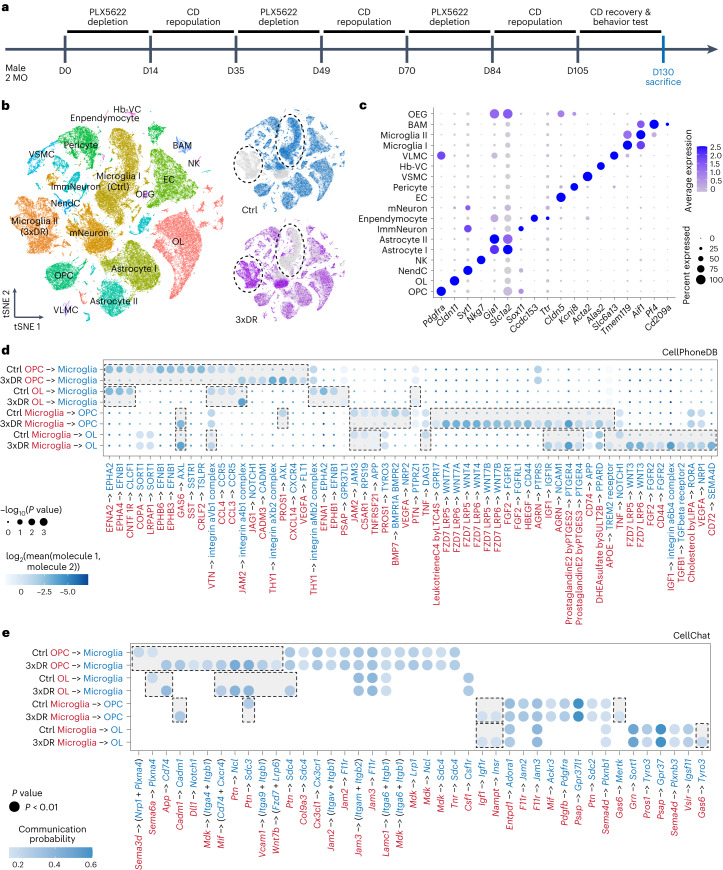
scRNA-seq characterizes the microglial crosstalk with OPCs and OLs in control and 3xDR brains. **a**, Scheme of the forced microglial turnover model and timepoints for experiments. Control mice are sex-matched and age-matched animals fed with CD for 130 d. **b**, tSNE plots of 11,952 control and 8,914 3xDR microglia, revealing an aged-like phenotype of 3xDR microglia. **c**, Dot plot showing the expression levels of well-known representative cell-type-enriched marker genes across all 16 cell types (18 populations). **d**, Significant ligand–receptor interactions predicted by CellPhoneDB. One-sided permutation test. **e**, Significant molecule–molecule interactions predicted by CellChat. One-sided permutation test. Hb-VC, hemoglobin-expressing vascular cell; ImmNeuron, immature neuron; mNeuron, mature neuron; NendC, neuroendocrine cell; OEG, olfactory ensheathing glia; VLMC, vascular and leptomeningeal cell; VSMC, vascular smooth muscle cell.

To further characterize the influence on aged microglia to other brain cells, we compared the transcriptome between 3xDR and age-matched control brains at single-cell resolution (Fig. 8a). We identified 16 cell types (18 populations) in control and 3xDR brains^42^ (Fig. 8b,c). Control microglia (Microglia I) and 3xDR microglia (Microglia II) located in distinct clouds (Fig. 8b). In contrast, other cell types did not show such a separation (Fig. 8b), further echoing that microglia are the major cell type directly influenced by 3xDR.

Our results indicate that 3xDR microglia dampen cognitive function via the myelination reduction. In line with this, the myelination signaling pathway was enriched in mature neurons by IPA (−log_10_*P* = 3.14). We thus investigated how 3xDR microglia influence OPCs and OLs by predicting the cell–cell crosstalk. We identified 60 ligand–receptor interactions differentially detected in 3xDR mice by CellPhoneDB (Fig. 8d). JAM2–JAM3, C5AR1–RPS19, TNF–DAG1 and TNF–NOTCH1 signaling pathways are associated with OL maturation and myelination. These pathways were enriched in the control but not the 3xDR brain (Fig. 8d), suggesting a dampened myelination support of aged-like 3xDR microglia. Moreover, we identified 42 significant transmitter–receiver interactions by CellChat, 16 of which were differentially involved between control and 3xDR (Fig. 8e). For the microglia and OPC interaction, *App*–*Cd74*, *Igf1*–*Igf1r* and *Gas6*–*Mertk* were differentially identified (Fig. 8e). For the microglia and OL interaction, *App*–*Cd74*, *Igf1*–*Igf1r* and *Gas6*–*Tyro3* were differentially identified (Fig. 8e). These molecule–molecule interactions are critical for cell survival, proliferation, differentiation and phagocytosis. The results provide clues for investigating how aged microglia regulate myelination.

In summary, our study systematically dissected the aging process of brain microglia. In addition, we developed an accelerated microglial turnover model, 3xDR. By this model, we are able to study the contribution of aged-like microglia in non-aged brain. Our results indicated that aged-like microglia per se contribute to cognitive decline (Supplementary Fig. 12).

## Discussion

In this study, we examined the mouse brain transcriptome in various adulthood stages by bulk RNA-seq. Based on this high-temporal-resolution microglial atlas, we identified ADEM genes that were continuously up-regulated or down-regulated during the aging process. The biological functions of ADEM genes are involved in cellular lipid and iron aggregation, pro-inflammatory cytokine and chemokine production and immune activation, which echoes previously identified deleterious roles of aged microglia. Increasing evidence indicates that aged microglia may exhibit pathological phenotypes during neurodegeneration^119^. We, therefore, compared ADEM and DAM genes and found 21 overlapping genes. Among these genes, *Axl*, *Spp1*, *Cst7*, *Fth1*, *Cybb*, *Lpl*, *Cd74* and *H2-D1* are well documented in both healthy aging and disease states^119–121^. These findings confirmed that ADEM genes were highly correlated with the etiology of neurodegenerative disorders. Furthermore, the integrative analysis of the microglial transcriptome and chromatin accessibility revealed that ADEM gene expression followed a stepwise chromatin opening pattern around the promoter region during the aging process. In addition, we found that CEBPβ and MEF2C in ADEM-accessible peaks exhibited aging-dependent activation. CEBPβ and MEF2C regulate microglial homeostasis and reactivity^66,68,70,71^, suggesting that they are potential key meditators of ADEM genes. Interestingly, we found a sex-specific microglial aging pattern. Whereas female microglia gradually aged in a stepwise manner, male microglia ‘suddenly’ became aged. The differences in the aging process may explain the sex differences in microglia^49,122–125^.

In contrast to the previous notion that aged microglia may display an exaggerated inflammatory response to LPS stimulation^75,126^, our results revealed that aged microglia in female mice exhibited attenuated immune reactivity upon systemic LPS challenge. As professional phagocytes in the brain, microglia respond to tissue damage and invading pathogens. The impaired migration and phagocytosis of aged microglia in pathological conditions cause the accumulation of cytotoxic molecules and prolonged neuroinflammation^119,127^. This could partially explain why aged brains are more vulnerable to neurological disorders. In our study, we harvested the brain tissue 2 h after LPS administration. Although previous studies from our and other groups showed that microglia are able to robustly respond to LPS challenge at the 2-h timepoint^41,77,88^, microglial reactivity might not reach a peak at this time. Therefore, when interpretating our results, it should be considered that the attenuated response might reflect the relatively early response upon LPS challenge.

The chromatin accessibility regulates gene expressions^60,68^. We found that chromatin accessibility around promoter regions was critical for regulating the dynamics of ADEM genes. Both natural aging and LPS challenge induced overt modifications of chromatin structure, leading to microglial reactivity and driving the expression of genes enriched in the inflammation pathway, such as IFN signaling pathway^65^. However, the chromatin modification patterns induced by aging and acute immune stimulation were distinct. LPS challenge led to a robust change in the accessibility of the first intron, whereas aging effects were mainly located in promoter regions (≤1 kb). Our results suggest distinct mechanisms underlying gene regulation in these two conditions. They also support the notion that natural aging and acute immune challenge result in distinct microglial phenotypes^128^.

Dysregulation of cell–cell communication is a hallmark of aging^50,90^. However, how aging influences the microglial crosstalk to other cells is not fully understood. We identified potential age-dependent cell–cell interactions and found that TNF, TGF-β, PDGF and VEGF signaling pathways were differentially involved in microglial crosstalk to astrocytes and ECs during the aging process. These pathways play critical roles in regulating immune response, cell proliferation and survival^5,129–131^. Several genes associated with neuroinflammation and oxidative stress were significantly up-regulated in aged ECs, indicating their roles in the overall inflammatory state of the aged brain. In aged astrocytes, genes involved in adaptive immunoregulation and the complement cascade were markedly elevated. Notably, *Occludin*, *Claudins* and *Cx4*, the key BBB component genes^97,98^, in astrocytes were not significantly altered during the aging process. The mechanism of BBB dysfunction in aged brain remains elusive. Some evidence suggests that it might be due to the dysfunction of ECs and astrocytes^94,95^. Our study suggests that the compromised BBB in the aged brain can be attributed to the inflammatory microenvironment and cell–cell crosstalk alteration.

The causality of aged microglia to brain function is poorly understood, because it is difficult to exclude complicated contributions from other aged non-microglial cell types. Hence, it is important to develop a model with aged microglia in the non-aged brain. CSF1R is exclusively expressed in brain microglia/macrophages and is necessary for microglial survival^132^. The inhibition of CSF1R selectively depletes 99% of brain microglia without directly affecting other cell types. When CSF1R inhibition is removed, residual microglia rapidly proliferate and recover to a normal density^41,45,102,133^. Thus, the depletion–repopulation approach accelerates microglial turnover. Through forced microglial turnover, 3xDR drives microglia to proliferate 23.71 times in a relatively short period and shortens the telomere. We thus used 3xDR to establish an accelerated microglial turnover model without directly influencing other non-myeloid cells in the brain, generating aged-like microglia in a non-aged milieu.

3xDR gives rise to aged-like phenotypes with morphological and transcriptional alterations in microglia. We found that 3xDR dampened learning and memory. Neuronal loss and neurogenesis deficits are undetected in 3xDR mice. Instead, the presence of aged-like microglia resulted in a significant reduction in myelin. Myelination is critical for cognitive functions^30,116^. Microglia facilitate myelination by phagocytosing excessive/apoptotic oligodendrocytes, myelin debris and dysfunctional myelin membranes. Previous studies demonstrated that the CX3CL1–CX3CR1 pathway and the TAM receptor tyrosine kinase MERTK mediate the microglial phagocytosis of myelin and oligodendrocytes^134,135^. Our scRNA-seq data showed that *Cx3cr1* and *Mertk* were down-regulated in aged-like 3xDR microglia. Dysregulated microglia phagocytosis may, thus, impede the OPC/OL differentiation, resulting in impaired myelination and cognitive decline. Therefore, converging evidences from the 3xDR model indicate that aged (or aged-like) microglia per se can impair cognitive function.

Notably, although non-myeloid brain cells are not directly influenced, they may be indirectly affected to some extent. For instance, astrocytes phagocytose the debris of dead microglia. During this process, astrocytes display a non-canonical reactivation phenotype^107^. The astrocytic phagocytosis of microglial debris is a physiological process during microglial turnover. The indirect influence of CSF1R inhibition to astrocytes might be natural. In addition, after microglia repopulate the whole brain, astrocyte reactivation markers resume to the homeostatic situation^45,102^, indicating that the indirect influence is transient. Future studies should investigate the potential influence of 3xDR to non-myeloid brain cells. Additionally, some studies suggested the non-microglial effects of CSFR1 inhibition^136^, albeit controversially^137^. The influence to BAMs should be considered.

As the CNS immune cells, microglia are sensitive to the microenvironment. First, a recent study indicated that the enzymatic digestion at 37 °C may result in ex vivo reactivation and affect the transcriptome. The influence can be minimized by either a Dounce homogenization at 4 °C or a cocktail of transcriptional and translational inhibitors^138^. The potential ex vivo reactivation should be taken into consideration. Second, most of mice in this study were deeply anesthetized by a cocktail of ketamine and xylazine before being euthanized. One study suggested an inhibitory effect of ketamine to the LPS-induced microglial reactivation^139^. The mRNA transcription usually takes 15–60 min to the peak^140–142^. The potential influence to the RNA-seq takes place after 15–60 min of ketamine administration. In contrast, animals were quickly euthanized by transcardial perfusion right after being deeply anesthetized (typically less than 5 min). The influence from ketamine would, thus, be minimal. Third, microglia exhibit heterogeneity in different brain regions^143–145^, putatively due to their divergent local microenvironment. More detailed investigations focusing on regional differences of microglial aging should be conducted in future studies.

## Methods

### Animals

C57BL/6J mice were either purchased from SPF Biotechnology Co., Ltd. or donated by Zhihui Huang at Hangzhou Normal University. P2Y12-CreER-GFP mice (*P2ry12*-p2A-CreER-p2A-EGFP) (ref. ^107^) were donated by Jiyun Peng at Nanchang University. TMEM119-GFP mice (C57BL/6-Tmem119em2(EGFP)Gfng/J, stock 31823) (ref. ^106^) were purchased from The Jackson Laboratory. All mice were housed in the Animal Facility at the Department of Laboratory Animal Science at Fudan University or the Shenzhen Institute of Advanced Technology, Chinese Academy of Sciences, under a 12-h light/dark cycle with food and water ad libitum. All animal experiments were conducted in accordance with the guidelines of the Institutional Animal Care and Use Committee of the Department of Laboratory Animal Science at Fudan University (202009001S, 202110005S and 2021JS-ITBR-002) and the Institutional Animal Care and Use Committee at the Shenzhen Institute of Advanced Technology, Chinese Academy of Sciences (SIAT-IACUC-190312-YGS-PB-A0576-01).

### Drug administration

To pharmacologically ablate brain microglia, mice were administered a PLX5622 (SYSE Bio, JP-2112)-formulated AIN-76A diet (1.2 g PLX5622 per kilogram of diet, formulated by SYSE Bio) ad libitum^41,45,102,146^. Control mice were fed with an AIN-76A CD. Because the microglial ablation efficiency by CSF1R inhibition is relatively lower in female mice^147^, we used male mice for this experiment.

To systemically challenge microglia, mice were treated with LPS (0.5 mg/kg body weight, Sigma-Aldrich, P4391) in PBS (treatment group) or PBS (vehicle control group) by intraperitoneal injection. Then, the mice were euthanized 2 h after administration.

### Brain tissue preparations

Mice were deeply anesthetized with a mixture of ketamine hydrochloride (100 mg/kg body weight) and xylazine (10 mg/kg body weight) by intraperitoneal injection. For histological experiments, animals were sequentially transcardially perfused with 0.01 M PBS and 4% paraformaldehyde (PFA) (Sigma-Aldrich, 441244) in 0.01 M PBS. Brains were then carefully harvested and post-fixed in 4% PFA in 0.01 M PBS at 4 °C overnight. For brain cell isolation, mice were transcardially perfused with 0.01 M PBS. Thereafter, the brain was immediately collected and excised on ice for subsequent procedures.

### Immunohistochemistry and image acquisition

After fixation, brains were dehydrated in 30% sucrose in 0.01 M PBS at 4 °C for 3–5 d. Then, brains were embedded in optimal cutting temperature compound (OCT) (Tissue-Tek). Brain samples were stored at −80 °C before cryosectioning. Tissue specimens including regions of interest were sectioned at a thickness of 30 μm or 15 μm with a Leica CM1950 cryostat, according to specific purposes.

After rinsing with 0.01 M PBS for three changes, brain sections were blocked and permeabilized with 4% normal donkey serum (NDS, Jackson ImmunoResearch, 017-000-121, lot: 153474) in 0.01 M PBS containing 0.3% Triton X-100 (PBST, Sigma-Aldrich, T8787) for about 2 hours at room temperature. Next, brain sections were incubated with primary antibodies with 1% NDS in PBST at 4 °C overnight. After washing primary antibodies using PBST, brain sections were stained with fluorescent dye-conjugated secondary antibodies with DAPI (1:1,000, Sigma-Aldrich, D9542) in 1% NDS in PBST at room temperature for 2 h. Thereafter, the samples were well rinsed three times before mounting with antifade mounting medium (Southern Biotech, Fluoromount-G, 0100-01).

The primary antibodies used in this study included rabbit anti-IBA1 (1:500, Wako, cat: 019-19741, lot: CAJ3125, SKM6526 and LEQ2171); goat anti-IBA1 (1:500, Abcam, cat: ab5076, lot: GR3381291-3 and GR3365012-2); goat anti-osteopontin/OPN (1:500, R&D Systems, cat: AF808, lot: BDO0720111); goat anti-AXL (1:200, R&D Systems, cat: AF854, lot: CTC0220081); rabbit anti-β galactosidase (1:2,000, Invitrogen, cat: A-11132, lot: 2304273); rabbit anti-Ki67 (1:250, Invitrogen, cat: MA5-14520, lot: VB2941291 and VE3003591); rat anti-Ki67 (1:1,000, Invitrogen, cat: 14-5698-82, lot: 2496198); rabbit anti-DCX (1:200, Abcam, cat: ab18723, lot: GR3274138-3); rabbit anti-PDGFRα (1:500, Cell Signaling Technology, cat: 3164S, lot: 02/2020-6); rabbit anti-NeuN (1:500, Abcam, cat: ab177487, lot: GR3275122-6); and rabbit anti-MBP (1:500, Abcam, cat: ab218011, lot: GR3299139-18) (Supplementary Table 8). All primary antibodies were diluted in 1% NDS in PBST.

The secondary antibodies used in this study included AF647 donkey anti-goat (Jackson ImmunoResearch, cat: 705-605-003, lot: 147708); AF488 donkey anti-chicken (Jackson ImmunoResearch, cat: 703-545-155, lot: 147805); AF488 donkey anti-mouse (Jackson ImmunoResearch, cat: 715-545-150, lot: 146643); AF488 donkey anti-goat (Jackson ImmunoResearch, cat: 705-545-003, lot: 145270); and Cy3 donkey anti-rabbit (Jackson ImmunoResearch, cat: 711-165-152, lot: 145020) (Supplementary Table 8). All secondary antibodies were diluted in 1% NDS in PBST.

Confocal images were acquired by using a Nikon AIR-MP confocal microscope with a solid-state laser. Lasers with wavelengths of 405 nm, 555 nm and 633 nm were used to excite the fluorophores. Plan-apochromat ×60 (oil) and ×40 objectives were used. *z-* stacked focal planes were acquired and maximally projected with Fiji. The brightness and contrast of the image were adjusted with Fiji if necessary.

### Preparation of single-cell suspension

For brain single-cell suspension (except for 3xDR versus control groups), brains without the cerebellum were minced into pieces and then dissociated in 8 U ml^−1^ papain lysis buffer containing 125 U ml^−1^ DNase I at 37 °C for 20 min with mild shaking. This process was terminated by adding 10% ovomucoid to the L15 culture medium. Thereafter, cell clusters were removed by filtering through a 70-μm nylon strainer (Falcon). Myelin and cell debris were removed through density gradient centrifugation in 37% Percol (Solarbio Life Science). Next, brain cells were thoroughly rinsed with EDTA-free FACS buffer (0.5% BSA in DPBS) before FACS and library preparation.

For single-cell suspension of 3xDR versus control groups, single-cell suspensions were prepared following the previous description with minor modifications^148^. In brief, brains without cerebellum and olfactory bulb from 3xDR and control mice were harvested and cut into 1-mm-thick sections using stainless steel brain matrices. Next, brain slides were incubated in 95% O_2_ and 5% CO_2_ bathed choline chloride solution (92 mM choline chloride, 2.5 mM KCl, 1.2 mM NaH_2_PO_4_, 30 mM NaHCO_3_, 20 mM HEPES, 25 mM glucose, 5 mM sodium ascorbate, 2 mM thiourea, 3 mM sodium pyruvate, 10 mM MgSO_4_·7H_2_O, 0.5 mM CaC_l2_·2H_2_O, 12 mM N-acetyl-l-cysteine) with 10 μM NBQX and 50 μM APV and 2% FBS (Gibco, l6140071) for about 30 min. After that, brain slides were digested in 20 U ml^−1^ papain and 100 U ml^−1^ DNase I (Worthington, lK003176) for 30 min at 37 °C, followed by incubation with 1 mg ml^−1^ protease (Sigma-Aldrich, P5147) and 1 mg ml^−1^ dispase (Worthington, LS02104) at 25 °C for 30 min with shaking. Next, brain slides were further tittered using a 1-ml pipette. Single-cell suspensions were then collected and filtered through 40-μm cell strainers (Falcon). The cell debris was removed by 30% Percoll (Millipore, P1644) in choline chloride solution. Finally, cells were resuspended in choline chloride solution for further experiments. Approximately 10^7^ cells were obtained from each brain. Approximately 24,000 cells for each mouse were loaded for 10x Genomics library preparation.

### FACS

Cells from young, middle-aged and aged mice were sorted by FACS as previously described^41,45^. In brief, the mixed brain cells were resuspended in FACS buffer. Then, mixed cells were stained with antibodies against CD11b (1:100, clone M1/70, BD Pharmingen, 557657) and CD45 (1:100, clone 30-F11, BD Pharmingen, 553080) in FACS buffer for 30 min on ice (Supplementary Table 8). Dead cells were labeled with 7-AAD (1:80, BD Pharmingen, 559925). Then, CD11b^+^ CD45^low^ 7-AAD^−^ microglia were collected by FACSAria III cell sorting (BD Biosciences). For brain cell scRNA-seq (except for 3xDR versus control groups), 7-AAD^−^ brain cells were collected. Harvested cells were then used for scRNA-seq, bulk RNA-seq and ATAC-seq.

### Magnetic-activated cell sorting

Mice were deeply anesthetized by isoflurane (open-drop) and perfused by cold normal saline (MeilunBio, MA0083). Then, the mouse brain was quickly harvested and minced on ice. After that, the mouse brain was dissociated by papain (8 U/ml) and DNaseI (125 U ml^−1^) for 30 min at 37 °C using gentleMAC Octo Dissociator with Heaters (Miltenyi Biotec, 130-096-427), followed by termination with trypsin inhibitor (1.5 mg/ml) in DMEM (Gibco, 14190144). Next, myelin and cell debris were removed from the single-cell suspension by Percoll density gradient centrifugation (30% v/v). Cell pellets were then resuspended in DPBS (Gibco, C11995500BT) containing 0.5% BSA (Beyotime, ST-025-5g) with 10% magnetic microbeads conjugated anti-mouse CD11b antibody (Miltenyi Biotec, 130-049-601) and incubated at 4 °C for 30 min. The cells were then washed and resuspended in DPBS containing 0.5% BSA. Microglia were enriched by flowing cell suspension through the LS column (Miltenyi Biotec, 130-042-401) attached to a QuadroMACS separator (Miltenyi Biotec, 130-091-051). After that, the column was washed twice by 5 ml of DPBS containing 0.5% BSA. Then, detained cells in the column were flushed out by 5 ml of DPBS containing 0.5% BSA. The enrichment process was repeated twice to obtain CD11b^+^ cells of higher purity. The purified cells were then used for DNA or RNA extraction immediately.

### RNA extraction and qPCR

Total RNA from isolated microglia was extracted with the RNAeasy Mini Kit (Qiagen, 74104) and FastPure Cell/Tissue Total RNA Isolation Kit V2 (Vazyme, RC112-01). Then, the total RNA was reverse transcribed into cDNA with the RT reagent kit (Takara, RR037), following the manufacturer’s instructions. qPCR was carried out using a one-step TB Green PrimeScript RT–PCR Kit (Takara, RR086) on a LightCycler 96 detection system (Roche). *Gapdh* was used as the internal control. The primers used in this study are listed in Supplementary Table 9.

### DNA extraction and telomere length measurement

Total DNA from isolated microglia was extracted by FastPure Blood/Cell/Tissue/Bacteria DNA Isolation Mini Kit (Vazyme, DC112), following the manufacturer’s instructions. The telomere length was assessed using Absolute Mouse Telomere Length Quantification qPCR Kit (ScienCell, M8918), according to the manufacturer’s protocol. Each PCR test contained about 2 ng of genomic DNA template. The telomere length was then calculated as reference telomere length × 2^((Cq_target sample telomere_ − Cq_reference sample telomere_) − (Cq_target sample single copy reference_ − Cq_reference sample single copy reference_))^149^.

### Primary microglia culture and Lipofectamine transfection for gene knock-down

Primary microglia were isolated and cultured as previously described^150^. In brief, a mixed glial cell culture was prepared from neonatal C57BL/6J mice and maintained for 10–21 d in DMEM containing 10 ml of 10% FBS (Gibco, 10100147) and 1% antibiotic-antimycotic (Gibco, 15240062) in a T75 flask. Microglia were collected by gentle shaking as the floating cells over the mixed glial cell culture. Then, primary microglia were transferred to a six-well plate for siRNA transfection experiments.

For siRNA transfection, primary microglia were pre-cultured to about 60% confluence in each six-well plate. Before siRNA transfection, primary microglia were cultured in DMEM without FBS for 4 h. Then, 50 nM Silencing *S100a8*, *S100a9* or negative control siRNA was transfected by 5 μl of Lipofectamine RNAiMAX (Thermo Fisher Scientific, 13778075), according to the manufacturer’s protocol. siRNA oligonucleotides targeting mouse *S100a8* and *S100a9* were synthesized by OBiO Technology.

### Open field test

The open field test was carried out as previously described^151^. In brief, mice were habituated in the behavior room in the presence of background white noise for at least 30 min before the behavior test. Individual mice were then placed in an open field arena (35 cm × 35 cm) by a blinded experimenter and allowed to freely explore the box for 20 min. The total distance, velocity and time spent in the center (center of the arena, 22 cm × 22 cm) were quantified using video tracking software (TopScan, CleverSys). All mice were handled gently to avoid stress in the experiment. Male mice were used in this behavior test.

### Y maze spontaneous test

The Y maze spontaneous test is a behavior test for measuring spatial working memory. This test was carried out in a Y-shaped maze composed of three arms (45 cm × 15 cm × 15 cm) at 120 °C angles from each other. The Y maze was placed 1 m above the ground. The movement of mice was recorded by an overhead camera. During the training process, the animal was introduced into one arm of the Y maze (start arm) and allowed to freely explore one of the other arms for 8 min (training arm). The behavior was analyzed by using EthoVision 11.5.1022 (Noldus). All mice were gently handled to avoid stress in the experiment. Correct alternation on Y maze is defined as the animal visited three consecutive arms clockwise or counterclockwise. Correct alternation rate was calculated by dividing the number of alternations by the number of possible alternations: ${\rm{correct}}\,{\rm{alternation}}\,{\rm{rate}}=\frac{{\rm{number}}\,{\rm{of}}\,{\rm{correct}}\,{\rm{alternation}}}{{\rm{number}}\,{\rm{of}}\,{\rm{total}}\,{\rm{arm}}\,{\rm{entry}}-2}\times 100$%. Male mice were used in this behavior test.

### Three-chamber social test

A three-chamber social test was conducted to assess social behavior and social interaction. This test included two sessions: a sociability session and a social novelty session. In the sociability session, mice were habituated to the testing room for at least 1 h before the behavior tasks. A subject mouse was introduced into the middle chamber for habituation for 5 min. Next, a stranger mouse (Stranger 1) was placed in the wire cage in one side chamber of the test apparatus. On the other side, there was a similar wire cage without a mouse. The subject mouse was allowed to freely explore all three chambers over a 5-min session. In the social novelty session, a novel stranger mouse (Stranger 2) was placed in the wire cage of the empty chamber while the familiar mouse (stranger 1) stayed in the previous chamber. The subject animal was allowed to explore for a 5-min session again. The time the subject mouse spent in each chamber and the time spent sniffing at each wire cage were analyzed by EthoVision 11.5.1022 (Noldus). All mice were gently handled to avoid stress in the experiment. Male mice were used in this behavior test.

### Morris water maze test

The Morris water maze test was performed to measure spatial learning as previously described^152^. In brief, a mouse was placed in a circular tank (1-m diameter) filled with opaque water. During the training period, the mouse was given the task of swimming to a visible platform located in the northwest quadrant of the tank. At the beginning of each trial, the mouse was placed in the water facing the wall of the tank in one of the four quadrants. Each mouse was allowed to find the platform in a period of 60 s. If the mouse was unable to find the platform within 60 s, it was guided and allowed to remain on the platform for 10 s. Each mouse was subjected to four trials every day with a 15-min interval during the training period. On day 6, the probe test was examined. The mouse was placed in the tank and allowed to find the hidden platform beneath the opaque water of the target quadrant. All swimming activities of the mouse were recorded by a video camera. The latency to reach the platform over four trials for each training day, the time spent in the target quadrant and the number of times the hidden platform was crossed were analyzed by using EthoVision 11.5.1022 (Noldus). All experiments were performed with gentle mouse handling to avoid stress in the experiment. Male mice were used in this behavior test.

### NOR test

The experiment was performed as previously described with minor modifications^153^. In brief, a 35-cm × 35-cm open field box was used as the experimental apparatus. Before the behavioral test, mice were habituated in the behavior room in the presence of background white noise for at least 30 min. At day 1, a mouse was habituated to the apparatus for free exploration for 20 min (pre-training, also as open field test). At day 2, the mouse was allowed to freely explore the apparatus with two identical objects for 5 min (stage I) and then was put back to the homecage. After a 5-min interval, the test mouse was placed into the arena again for 5 min (stage II). Then, the mouse was put back to the homecage for 10 min. For the test phase (stage III), one of the two objects in the open field box was replaced by a new object. The mouse explored the open field with one familiar object and one novel object for 3 min. Times spent in each object were analyzed by EthoVision 11.5.1022 (Noldus). All mice were gently handled to avoid stress in the experiment. Male mice were used in this behavior test.

### Analysis of bulk RNA-seq data

RNA-seq data were filtered with SOAPnuke 1.5.2 by removing reads containing sequencing adapters, reads with a low-quality base ratio (base quality ≤5) greater than 20% and reads with an unknown base (‘N’ base) ratio ≥5%, as previously described^154^. Thereafter, clean reads were obtained and stored in FASTQ format. The clean reads were then mapped to the reference genome mm10 using HISAT2 2.0.4. Bowtie2 2.2.5 was applied to align the clean reads to the mouse reference coding gene set. Gene expression levels were determined with RSEM 1.2.12.

Downstream data analysis and visualization were performed with RStudio 3.6.3. Batch effect correction of all samples (including female and male samples) was conducted using the ComBat_seq function in SVA 3.35.2. Genes with an average expression level of fewer than 10 fragments per kilobase of transcript per million mapped reads (FPKM) were excluded from further analysis. Correlation among samples was calculated by the cor function. PCA was based on a library size-normalized count matrix using the prcomp function, and the results were visualized using the fviz_pca_ind function of factoextra 1.0.7.

DEG analysis was performed using EdgeR 3.28.1 by using quasi-likelihood (QL) *F*-tests. Genes with *P* < 0.05 and |log_2_FC| > log_2_1.5 were determined to show statically significant differences in two-group comparison, whereas genes with an adjusted *P* < 0.05 and at least one |log_2_FC| > log_2_1.5 were determined to show statically significant differences in comparisons of more than two groups. GO analysis was performed using the enrichGO function of the clusterProfiler 3.14.3 package, where categories with a false discovery rate (FDR) ≤ 0.05 were considered significantly enriched; categories were simplified using the ‘simplify’ function with a cutoff value = 0.7, and visualization was performed using the dotplot and emapplot functions in clusterProfiler 3.14.3. DEGs were also subjected to IPA for functional analysis using the Ingenuity Knowledge Base (Qiagen Bioinformatics). The FC and FDR values of each gene were used to perform the core analysis, and pathways enriched with an adjusted *P* < 0.05 were considered significantly enriched. Venn diagrams, heat maps and volcano plots were visualized by using VennDiagram 1.6.20, pheatmap 1.0.12 and EnhancedVolcano 1.4.0, respectively.

To identify ADEM genes, we examined the average gene expression levels of each gene in females and males across their lifespan and compared the relative gene expression levels between neighboring timepoints. DEGs that were continuously up-regulated or down-regulated in at least five adjacent groups were defined as ADEM genes.

### Analysis of scRNA-seq data

Sample demultiplexing, barcode processing and single-cell 3′ gene counting were carried out with Cell Ranger 2.0.1 or 5.0.0 (10x Genomics). The 10-bp transcripts/unique molecular identifier (UMI) tags were extracted from the reads. Cell Ranger mkfastq with bcl2fastq 2.0.1 was applied to demultiplex raw base call files from the sequencer into sample-specific FASTQ files. Additionally, Cell Ranger-compatible references were produced based on both genome sequences and transcriptome GTF files by using Cell Ranger ‘ref’. These FASTQ files were aligned to the reference genome mm10 with Cell Rranger ‘count’ by using STAR 2.5.3. Aligned reads were then filtered for valid cell barcodes and UMIs to generate filtered gene–barcode matrices.

Next, gene–barcode matrices were analyzed with Seurat 3.2.0. For quality control, low-quality cells (detected >4,000 genes, <200 genes and >5% mitochondrial genes) were discarded. Filtered data were further normalized by the NormalizeData function and scaled by ScaleData. For scRNA-seq, data from female microglia were analyzed by using RunPCA with VariableFeatures, and the top 20 principal components (PCs) of the datasets were used for clustering and tSNE/uniform manifold approximation and projection (UMAP) visualization. For microglial transcriptome analysis, clusters with high expression of *S100a4*, *Cd79b*, *Nkg7*, *S100a9* and *Camp* were regarded as non-microglia clusters and excluded from microglial single-cell analysis. DEGs among groups of cells were identified using FindAllMarkers or FindMarkers with parameters of min.pct = 0.1 and thresh.use = 0.25 (Wilcoxon rank-sum test). Genes with a |log_2_FC| > 0.25 and *P* < 0.05 were included and defined as DEGs. These DEGs were further used for GO and IPA analyses as described above.

For ADEM gene signature score analysis, up-regulated and down-regulated ADEM DEGs with gene signatures were used to determine module scores by AddModuleScore and visualized by FeaturePlot and VlnPlot.

For scRNA-seq of PBS-treated and LPS-treated microglia, the top 40 PCs were used for clustering and t-SNE/UMAP visualization. For brain cell scRNA-seq data, the top 50 PCs were used for clustering and t-SNE/UMAP visualization. For the scRNA-seq of forced microglial turnover, the top 30 PCs were used for clustering and t-SNE/UMAP visualization. DEG analyses were performed as described above.

To identify different brain cell types, we used multiple cell-type-specific or enriched marker genes as previously described^42^. In brief, these genes included, but were not limited to, *P2ry12*, *Tmem119*, *Hexb* and *Aif1* for microglia; *S100b*, *Slc1a2* and *Aldh1l1* for astrocytes; *Nkg7* for NK cells; *Tubb3* and *Dcx* for neurons; *Vtn* for pericytes; *Abcg2* for ECs; *Acta2* for vascular smooth muscle cells; *Ttr* for choroid plexus epithelial cells; *Alas2* for red blood cells; *Slc47a1* for age-associated B cells (ABCs); *S100a9* for neutrophils; *Pdgfra* for OPCs; *Mbp*, *Mag* and *Mog* for oligodendrocytes; *Plac8* and *Ccr2* for monocytes; and *Ccdc153* for ependymocytes.

### Analysis of cell–cell interactions

CellPhoneDB version 2.0 was used to assess putative interactions of microglia–astrocyte and microglia–EC following the standard workflow^99,100^. The input files were generated from the Seurat object. For 3-month and 24-month data, the statistical analysis of CellPhoneDB was run individually in the normal mode with default parameters. Dot plots and heat maps were generated by dot_plot and heatmap_plot functions.

CellChat was used to predict the cell–cell communication with default recommended parameters following the ‘Comparison analysis of multiple datasets using CellChat’ tutorial^101^. CellChat objects were generated from the Seurat object. Then, the data were pre-processed by subsetData, identifyOverExpressedGenes and identifyOverExpressedInteractions functions with default parameters. Communication probability and communication network were computed by computeCommunProb function. Then, compareInteractions was used for comparing the interaction strength between different ages/groups. Up-regulated and down-regulated signaling ligand–receptor pairs were identified by comparing communication probabilities with the netVisual_bubble function. The CellChatDB mouse was used for analysis.

### Pseudotime trajectory

Pseudotime trajectory analysis was conducted by Monocle 3 (0.2.2) (refs. ^155,156^). After the construction of the CDS object, cells with microglial identity were stored for further analysis. UMAP was then applied for dimensional reduction using reduce_dimension in Monocle 3 with parameter reduction_mothod = ‘UMAP’. Pseudotime information and trajectory were generated by learn_graph and order_cells. root_cells was selected according to the real-time situation. Cells were assigned a specific pseudotime value based on their projection on the UMAP graph in the learn_graph function. Then, the results were visualized using the plot_cells and plot_genes_in_pseudotime functions of Monocle 3.

### Analysis of ATAC-seq data

For ATAC-seq, we pooled microglia from five mice into two libraries (2–3 mice for each library). The paired-end adapter reads were trimmed using Cutadapt 2.8 with the parameters cutadapt -j 0 -m 20 -q 20. Reads were then aligned to the reference mouse genome mm10 using Bowtie2 2.4.1 with parameters bowtie2 -p 8 -X 1000–dovetail–very-sensitive. The duplicate reads were excluded using the samtools markdup command. Reads aligned to the mitochondrial genome and reads aligned to ENCOD blacklisted regions were filtered by Genrich with the default parameters. Then, the remaining read peaks were determined using Genrich. Heat maps of coverage at TSSs were generated using computeMatrix with the parameters computeMatrix reference-point–referencePoint TSS -b 1000 -a 1000 -R and plotHeatmap functions in deepTools 3.5.1. Peaks of interest were visualized with make_tracks_file and pygenometracks. Chromatin location information was obtained by using IGV 2.8.0, with the reference genome mm10.

Next, the ATAC-seq peak count matrix was calculated by using Rsubread 2.0.1. Differentially accessible regions were identified by DESeq2. These data were then analyzed and visualized with IGV 2.8.0. Differentially accessible chromatin regions were further used for location annotation analysis. In brief, the differential open chromatin regions were annotated using annotatePeak with the parameters TxDb =TxDb.Mmusculus.UCSC.mm10.knownGene and annoDb = ‘org.Mm.eg.db’ in ChIPseeker 1.22.1. The results were then visualized by using plotAnnoBar.

For TF motif identification, ADEM genes and DEGs between samples were used as inputs by HOMER findMotifsGenome.pl with the default parameters. Correlation analysis of TFs and genes was performed using the aforementioned method. Heat maps and volcano plots were generated by pheatmap 1.0.12 and EnhancedVolcano 1.4.0, respectively. chromVAR 1.8.0 and chromVARmotifs 0.2.0 were used to quantify TF activity in *n* = 332 motifs from the HOMER database. The input fragment count matrix was generated by Rsubread 2.0.1. Deviation values were then generated by computeDeviations and visualized in GraphPad Prism 8.

### Microglial morphology analysis

For microglial morphology analysis, microglia from cortex and hippocampus were visualized by IBA1 and imaged with a Nikon AIR-MP confocal microscope and then subjected to Sholl analysis as previously described^41,157^. In brief, *z*-stack confocal images of microglia were transformed to 8-bit binary and skeletonized images. Next, skeletonized images were quantified with the Sholl analysis tool of Fiji. Concentric circles were drawn with center on the soma, beginning at 5-μm radius and increasing by 5 μm for each cycle. The number of intersections for each branch and each increasing circle was calculated. About 10–12 microglia were analyzed for each mouse, 100 microglia in total. The microglial senescence index was used to estimate microglial senescence in previous studies^105^. This index was calculated as the ratio of the microglial density to the morphological complexity (the area under the curve of Sholl analysis). For immunoreactivity quantification, pixels of target protein or RNA fluorescent signals were normalized and calculated by Fiji^41,45^. Statistics and graphics were generated using Prism 8.3.0 and R 3.6.1. The unpaired *t*-test was used to compare data between two groups. Data are presented as mean ± s.d. unless otherwise stated. Statistical significance was set as *P* < 0.05.

### Microglial density analysis

For microglial density analysis, IBA1^+^ microglia were imaged by an Olympus VS200 microscope. In brief, microglia numbers were quantified in a hemisphere of coronal brain section (typically at bregma −1.22 mm to −2.18 mm). The cell density is the cell number divided by the hemisphere area.

### Statistics and reproducibility

The statistical approaches are indicated in the figure legends. No statistical methods were used to pre-determine sample sizes, but our sample sizes are similar to those reported in previous publications^41,45,83,102,107,146,157^. Mice were not randomized for the age-related experiments because mice of different ages were acquired for experiment. For the LPS challenge and 3xDR-related experiments, block randomization was performed on cages of mice such that an approximate number of mice per cage were assigned to each experimental group. Data distribution was assumed to be normal, but this was not formally tested. Collection and analysis of bulk RNA-seq, scRNA-seq and ATAC-seq data were not performed blinded to the conditions of the experiments. Collection of behavior experiment data was blinded except for the old mice, because old mice look different than younger mice. No data were excluded from the analyses.

### Reporting summary

Further information on research design is available in the Nature Portfolio Reporting Summary linked to this article.

### Supplementary information

Supplementary Information Supplementary Figs. 1–12.

Reporting Summary

Supplementary Table 1 Information on DEGs, ADEM genes, GO biological functions and ingenuity pathways of microglial bulk RNA-seq.

Supplementary Table 2 Information on peak counts for ADEM genes and motifs associated with ADEM genes.

Supplementary Table 3 Information on DEGs in each scRNA-seq cluster, GO terms of C13–C16, DEGs of LPS-treated versus PBS-treated microglia at each age, *z*-score data at each age and some biological functions.

Supplementary Table 4 Information on differentially accessible chromatin regions of LPS-treated versus PBS-treated microglia at each age.

Supplementary Table 5 Information on markers used for cell type identification, DEGs in astrocytes, ECs and microglia and GO for astrocytes, ECs and microglia.

Supplementary Table 6 Information on DEGs and ingenuity pathways of ECs and astrocytes.

Supplementary Table 7 Information on DEGs and ingenuity pathways in 3xDR and control microglia.

Supplementary Table 8 Antibodies used in this study.

Supplementary Table 9 Primers used in this study.

Supplementary Data 1 Statistical source data related to Supplementary Fig. 1.

Supplementary Data 2 Statistical source data related to Supplementary Fig. 3.

Supplementary Data 3 Statistical source data related to Supplementary Fig. 5.

Supplementary Data 4 Statistical source data related to Supplementary Fig. 8.

Supplementary Data 5 Statistical source data related to Supplementary Fig. 11.

### Source data

Source Data Fig. 1 Exact values in scatter plot, full enrichment results and genes list in Venn plot.

Source Data Fig. 2 Percentage of each cluster in Fig. 2c.

Source Data Fig. 3 Unprocessed source data in Fig. 3.

Source Data Fig. 4 Unprocessed source data in Fig. 4.

Source Data Fig. 5 Statistical source data in Fig. 5.

Source Data Fig. 6 Statistical source data in Fig. 6.

Source Data Fig. 7 Statistical source data in Fig. 7.

Source Data Extended Data Fig. 1 Statistical source data in Extended Data Fig. 1.

Source Data Extended Data Fig. 2 Statistical source data in Extended Data Fig. 2.

Source Data Extended Data Fig.3 Statistical source data in Extended Data Fig. 3.

Source Data Extended Data Fig. 6 Statistical source data in Extended Data Fig. 6.

Source Data Extended Data Fig. 7 Statistical source data in Extended Data Fig. 7.

Source Data Extended Data Fig. 8 Statistical source data in Extended Data Fig. 8.

Source Data Extended Data Fig. 9 Statistical source data in Extended Data Fig. 9.

Source Data Extended Data Fig. 10 Statistical source data in Extended Data Fig. 10.

## Data Availability

The data that support the findings of this study are available from the corresponding author within 3 months upon reasonable request. Bulk RNA-seq data are available in the Gene Expression Omnibus (GEO) with accession code GSE208386. scRNA-seq data of the microglia of 3-month-old, 14-month-old and 24-month-old PBS-treated and LPS-treated mice are available in the GEO with accession code GSE207932. scRNA-seq data of control and 3xDR microglia are available in the GEO with accession code GSE207948. scRNA-seq data of brain cells of 3-month-old and 24-month-old mice are available in the GEO with accession code GSE208292. scRNA-seq data of control and 3xDR brain cells are available in the GEO with accession code GSE226286. ATAC-seq data are available in the GEO with accession code GSE208346. Processed data are available in Supplementary Tables 1–7. The source data of statistical results are provided with this paper. To disseminate these data to the community, we generated an interactive website for searching the data (http://www.microgliatlas.com).
