# Publisher Correction: Transcriptional and epigenetic decoding of the microglial aging process

**DOI:** 10.1038/s43587-024-00571-w

**Published:** 2024-01-10

**Authors:** Xiaoyu Li, Yuxin Li, Yuxiao Jin, Yuheng Zhang, Jingchuan Wu, Zhen Xu, Yubin Huang, Lin Cai, Shuai Gao, Taohui Liu, Fanzhuo Zeng, Yafei Wang, Wenxu Wang, Ti-Fei Yuan, Hengli Tian, Yousheng Shu, Feifan Guo, Wei Lu, Ying Mao, Xifan Mei, Yanxia Rao, Bo Peng

**Affiliations:** 1 Department of Neurosurgery, Jinshan Hospital, Institute for Translational Brain Research, State Key Laboratory of Medical Neurobiology, MOE Frontiers Center for Brain Science, Innovative Center for New Drug Development of Immune Inflammatory Diseases, Ministry of Education, Fudan University, Shanghai, China; 2 Department of Neurology, Zhongshan Hospital, Department of Laboratory Animal Science, MOE Frontiers Center for Brain Science, Fudan University, Shanghai, China; 3 Department of Neurosurgery, Huashan Hospital, Fudan University, Shanghai, China; 4 Shenzhen Institute of Advanced Technology, Chinese Academy of Sciences, Shenzhen, China; 5 Department of Neurology, Shanghai Sixth People’s Hospital Affiliated to Shanghai Jiao Tong University School of Medicine, Shanghai, China; 6 Department of Orthopedics, The First Affiliated Hospital of Jinzhou Medical University, Jinzhou, China; 7 Shanghai Key Laboratory of Psychotic Disorders, Shanghai Mental Health Center, Shanghai Jiao Tong University School of Medicine, Shanghai, China; 8 Co-Innovation Center of Neurodegeneration, Nantong University, Nantong, China

**Keywords:** Microglia, Neuroimmunology, Ageing

Correction to: *Nature Aging* 10.1038/s43587-023-00479-x, published online 11 September 2023.

In the version of the article initially published, the number of a grant from the National Natural Science Foundation of China was listed in the Acknowledgements as 3217095 instead of 32170958. The error has been corrected in the HTML and PDF versions of the article.

