# Supplementary material for: Transcriptional and epigenetic decoding of the microglial aging process
Source: Nat Aging. 2023 Sep 11;3(10):1288–311. doi: 10.1038/s43587-023-00479-x (PMC10570141; doi:10.1038/s43587-023-00479-x)
Supplement: Supplementary file 1 — Supplementary Figs. 1–12. [file 43587_2023_479_MOESM1_ESM.pdf]

nature aging

Resource https://doi.org/10.1038/s43587-023-00479-x

Transcriptional and epigenetic decoding of the microglial aging process

In the format provided by the authors and unedited

Supplementary figures

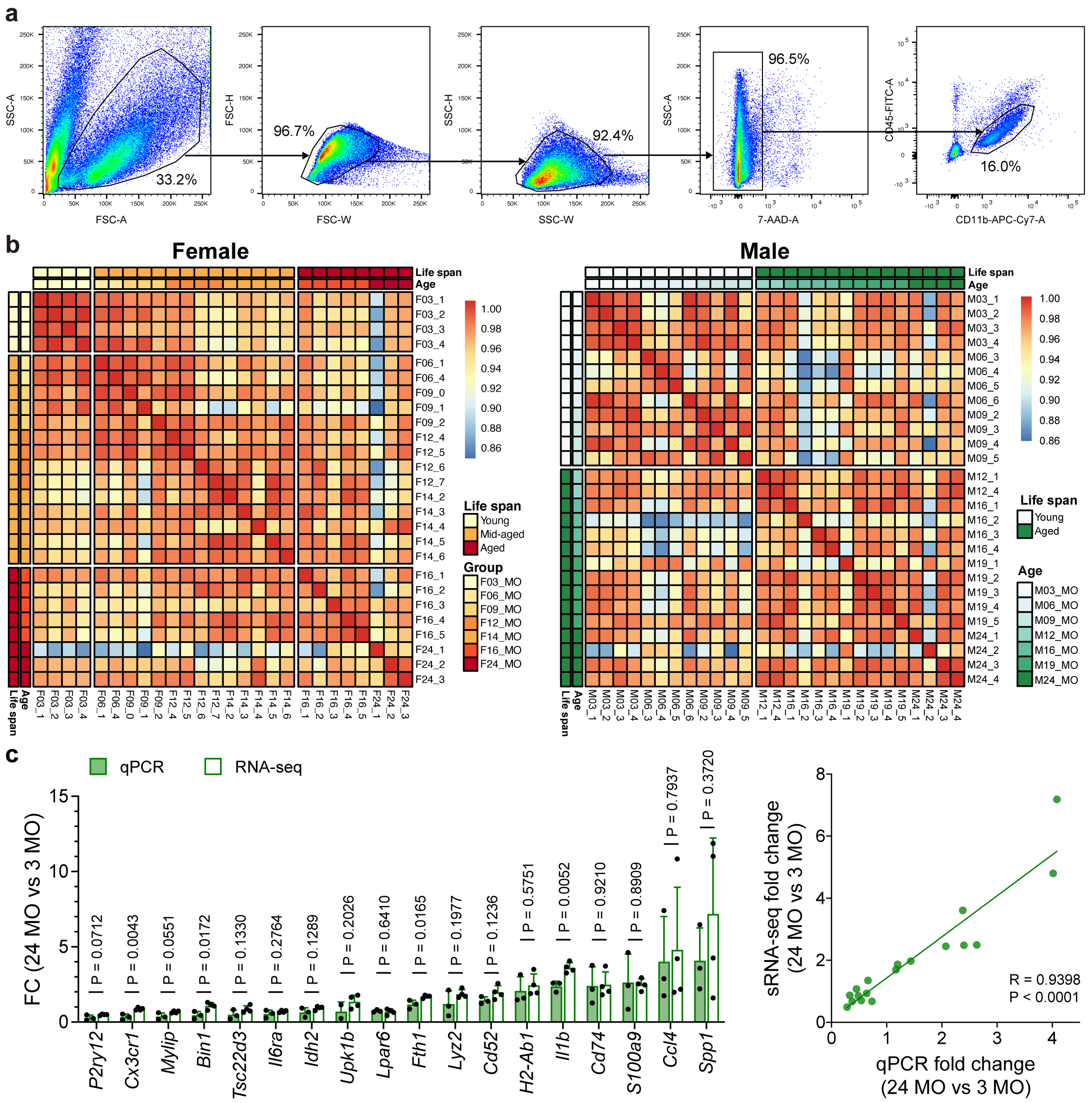

Supplementary Figure 1 Gating strategy and validation of microglia bulk RNA-seq. **(a)** FACS gating strategy for harvesting CD11b^{+} CD45^{low} cells (microglia).

(b) Heatmap of Pearson correlation across samples; each column indicates one biological sample. Pearson correlation coefficients are represented in color as indicated on the scale on the left, and samples in the same age or life span were annotated with the same color above the heatmap.

(c) Bulk RNA-seq results were validated by qPCR. Linear regression of FC of selected genes between scRNA-seq and qPCR validated the sequencing. Data are presented as mean ± SD. N = 3 to 4 mice for each group. Two-tailed independent t test (left) and

linear regression (right).

MO: month-old; FC: fold change.

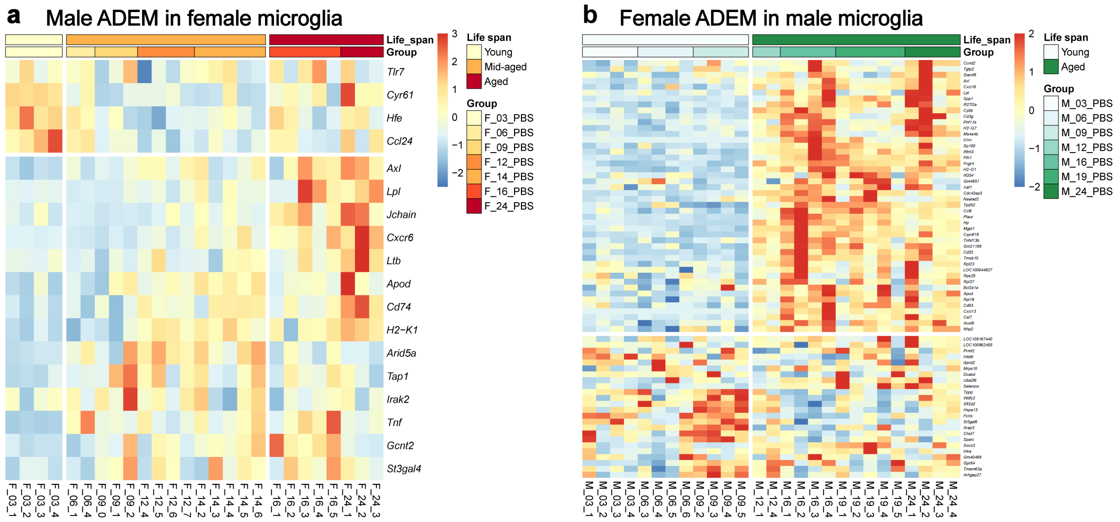

Supplementary Figure 2 Male ADEM in female microglia and female ADEM in male microglia.

(a) Most of male ADEM genes exhibited up- and down-regulation trends with stepwise transitions in female microglia, though the trends were not as prominent as the trends of female ADEM in female microglia.

(b) Most of female ADEM genes in male microglia presented a precipitous switch between young and aged stages.

ADEM: age-dependent microglia.

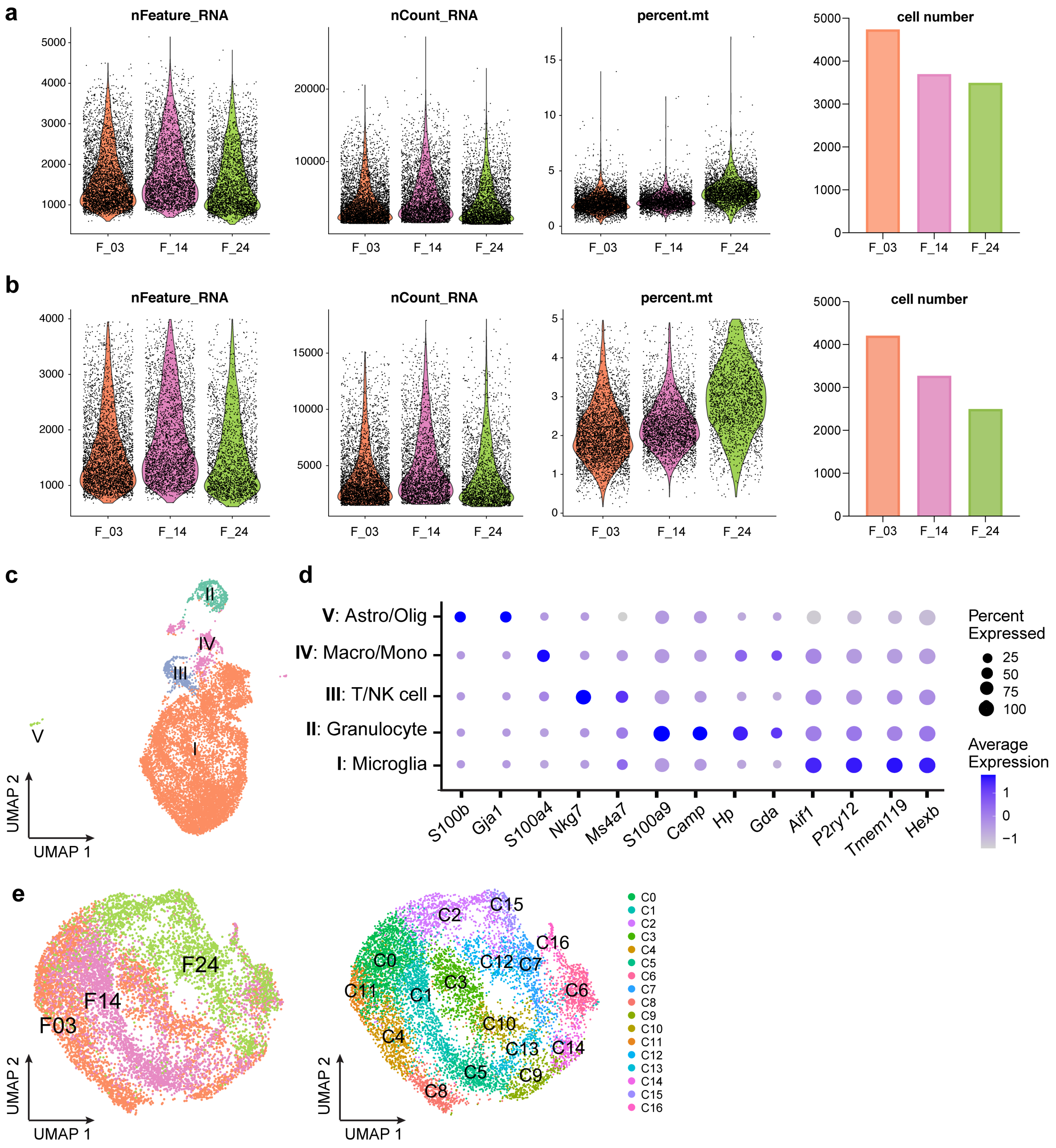

Supplementary Figure 3 Quality control and cell type classification for microglial scRNA-seq.

(a-b) Quality control for scRNA-seq before (a) and after (b) removal of low-quality, doublets and putatively dead cells. Violin plots showing the number of genes detected in each cell (nFeature_RNA), total number of molecules detected within a cell (nCount_RNA) and the distribution of mitochondrial genes in each cell (percent.mt) among the 3 groups. Bar plots showing the number of cells in the 3 groups. N = 5 mice for each group. 4,742 young (3 MO), 3,697 middled-aged (14 MO) and 3,495 aged (24 MO) microglia in (a). 4,207 young (3 MO), 3,272 middled-aged (14 MO) and 2,497 aged (24 MO) microglia in (b).

(c) UMAP plot illustrates that harvested cells are divided into 5 clouds, including microglia, granulocytes, T cells, NK cells, macrophages, monocytes, astrocytes and oligodendrocytes, and cells are colored according to the cell type.

(d) Dot plot shows the expression levels of well-known representative cell-type-enriched marker genes across all 5 cell types.

(e) UMAP plots of young (3 MO), middled-aged (14 MO) and aged (24 MO) microglia. Cells were divided into 17 clusters (C0-C16) by unsupervised classification, and cells were colored according to age (left panel) and cluster (right panel).

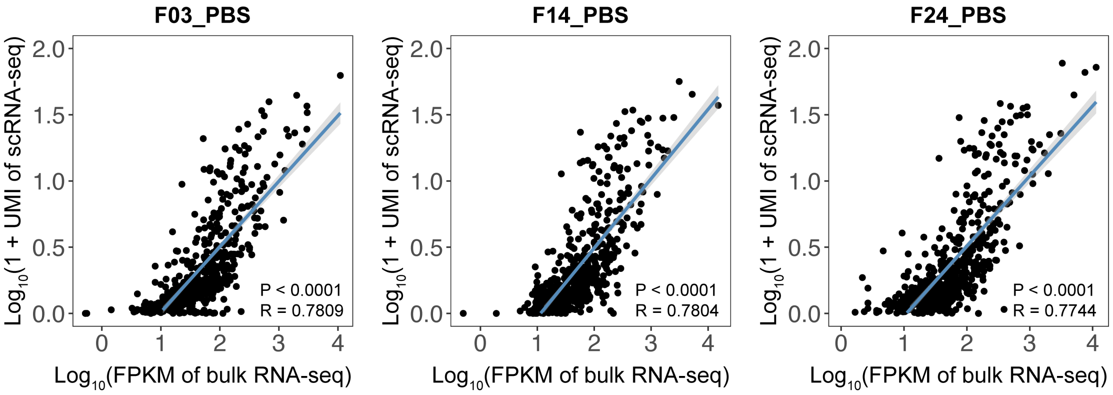

Supplementary Figure 4 Linear regressions verify that the scRNA-seq results are correlated with bulk RNA-seq.

Scatter plot showing the correlation of expression levels between scRNA-seq (y-axis) and bulk RNA-seq (x-axis).

The gray shadow represents the 95% confidence interval.

F03: 3-month-old female, and so on in a similar fashion.

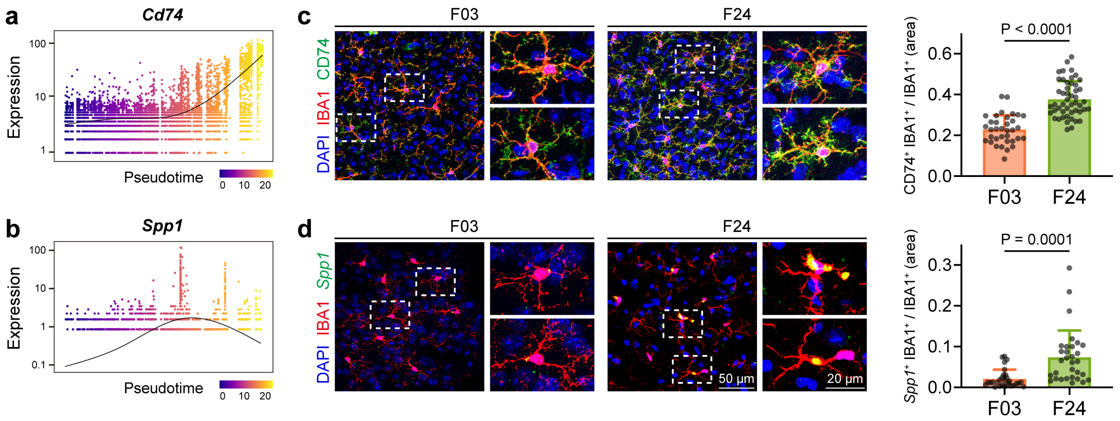

Supplementary Figure 5 Validation of *Cd74* and *Spp1* between young and aged microglia.

(a-b) *Cd74* and *Spp1* expressions versus pseudotime.

(c) Immunostaining shows that CD74 protein level increases in aged microglia. N = 35 cells for F03 and 49 cells for F24.

(d) RNAscope shows that *Spp1* mRNA level increases in aged microglia. N = 29 cells for F03 and 31 cells for F24.

Data are presented as mean ± SD. Two-tailed independent t test.

MO: month-old; F03: 3-month-old female, and so on in a similar fashion.

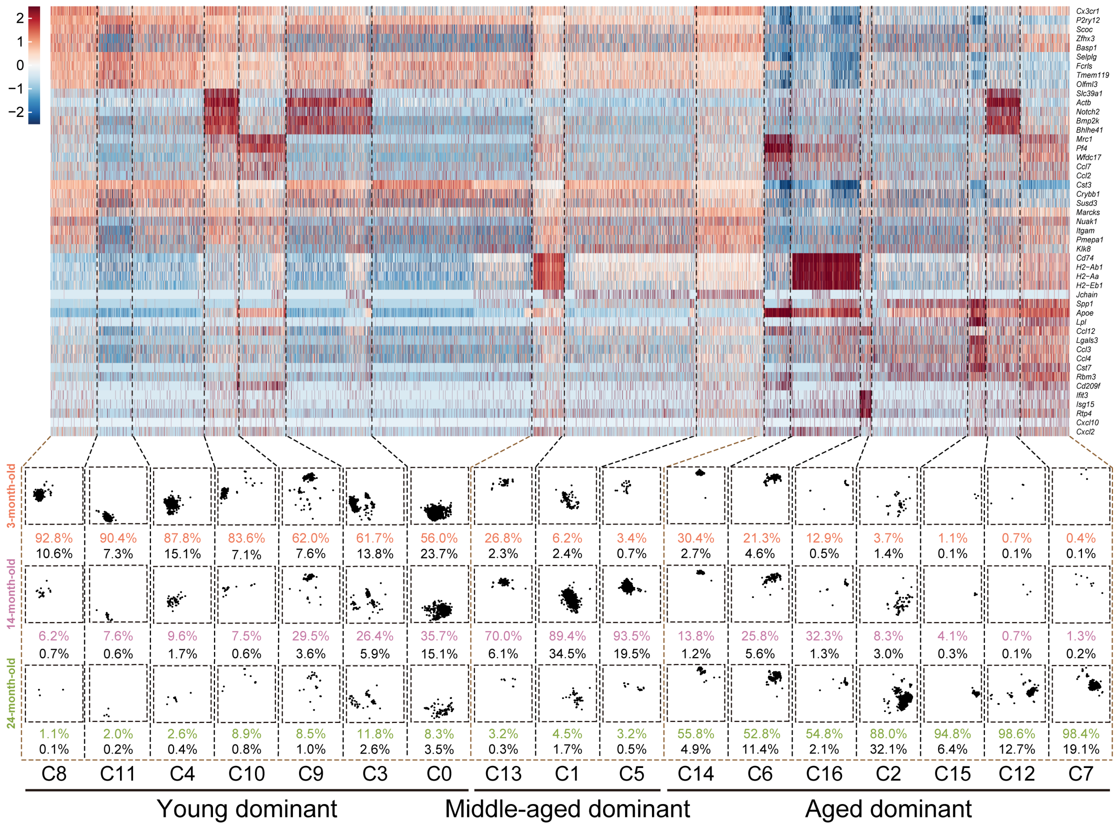

Supplementary Figure 6 Heatmaps and tSNE plots representing the top DEGs and cell percentages in each cluster.

Heatmap showing the top 5 highly expressed genes of clusters identified in Figure 2b, tSNE plot showing the expression pattern of each cluster. Colored percentage: cell percentage in each cluster; black percentage: cell percentage at each age.

Colored percentage: cell percentage in each cluster; black percentage: cell percentage at each age.

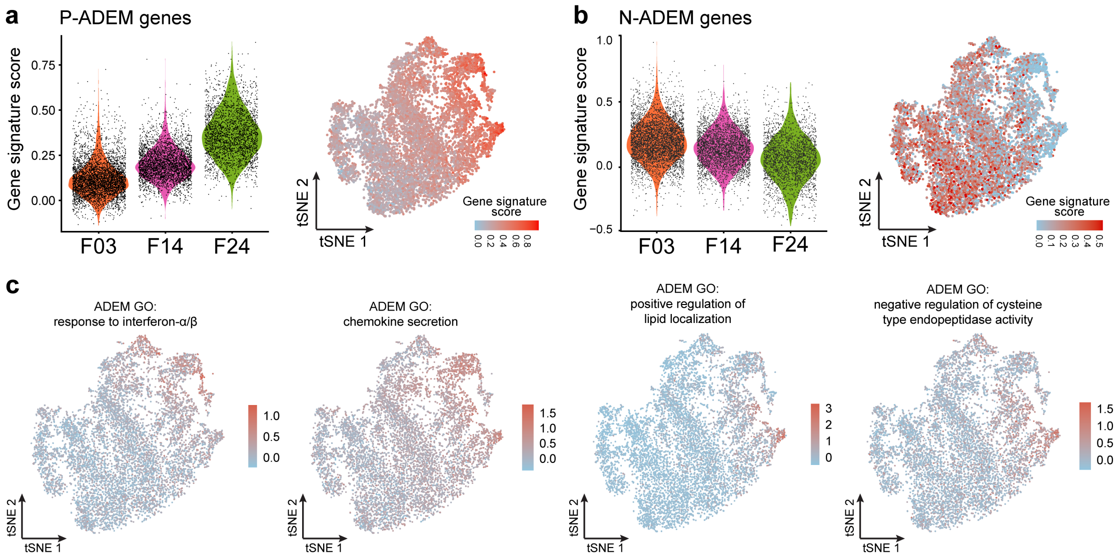

Supplementary Figure 7 Gene signature scores of ADEM are correlated with age.

(a-b) Gene signature scores of P-ADEM (a) and N-ADEM (b). Violin plot and feature plot showing that the gene signature score of P-ADEM genes is positively correlated with age and that the gene signature score of N-ADEM genes is negatively correlated with age.

(c) Gene signature scores of ADEM genes related to interferon response, chemokine secretion, positive regulation of lipid localization and negative regulation of cysteine type endopeptidase activity.

N = 5 mice for each group. 4,207 young (3 MO), 3,272 middled-aged (14 MO) and 2,497 aged (24 MO) microglia are harvested. ADEM: age-dependent microglia; GO: Gene Ontology.

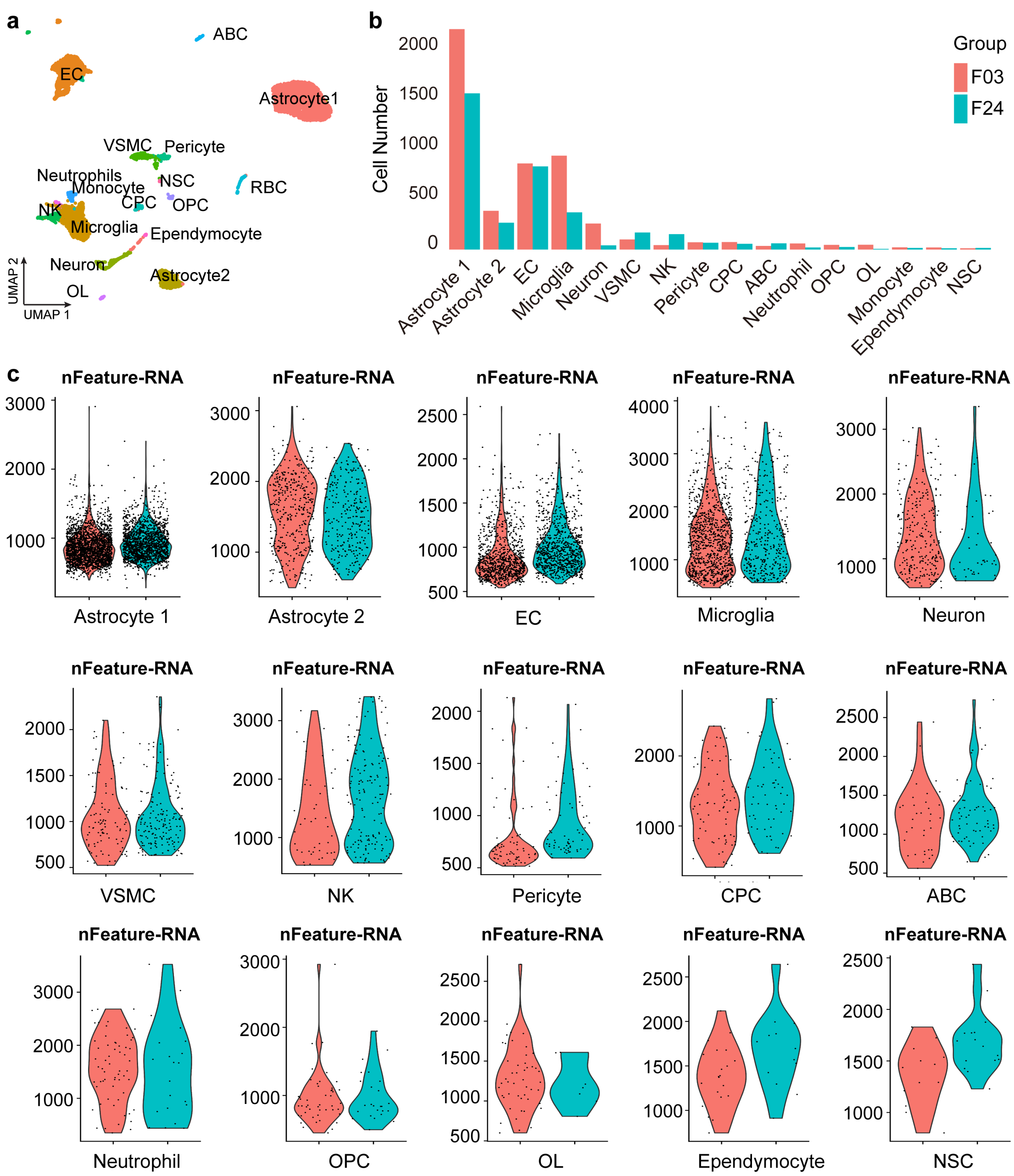

Supplementary Figure 8 Quality control of the whole brain cell scRNA-seq.

(a) UMAP plot illustrating the cell classifications in the brain cell scRNA-seq analysis.

(b) Cell numbers of each cell population.

(c) Violin plots showing detected gene numbers in each cell population, reflecting the quality of scRNA-seq.

N = 5 mice for each group. 4,326 young (3 MO) and 2,762 aged (24 MO) brain cells are harvested. EC: endothelial cell; VSMC: vascular smooth muscle cell; OL: oligodendrocyte; OPC: oligodendrocyte precursor cell; ABC: arachnoid barrier cell; NSC: neural stem cell; CPC: choroid plexus epithelial cell; NK: natural killer cell.

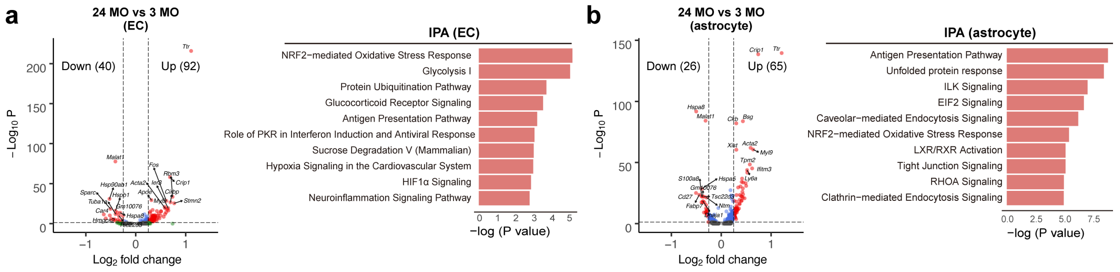

Supplementary Figure 9 Volcano plots and Ingenuity Pathway Analyses of 24- vs. 3-month-old ECs and astrocytes.

(a) Volcano plots (left) showing the age-related DEGs (24 vs. 3 months of age) of ECs and functionally annotated by IPA (right).

(b) Volcano plots (left) showing the age-related DEGs (24- vs. 3-months-old) of astrocytes and functionally annotated by IPA (right).

Non-parametric Wilcoxon rank sum test (volcano plot) and accumulative hypergeometric test (IPA). MO: month-old; EC: endothelial cell; IPA: Ingenuity Pathway Analysis.

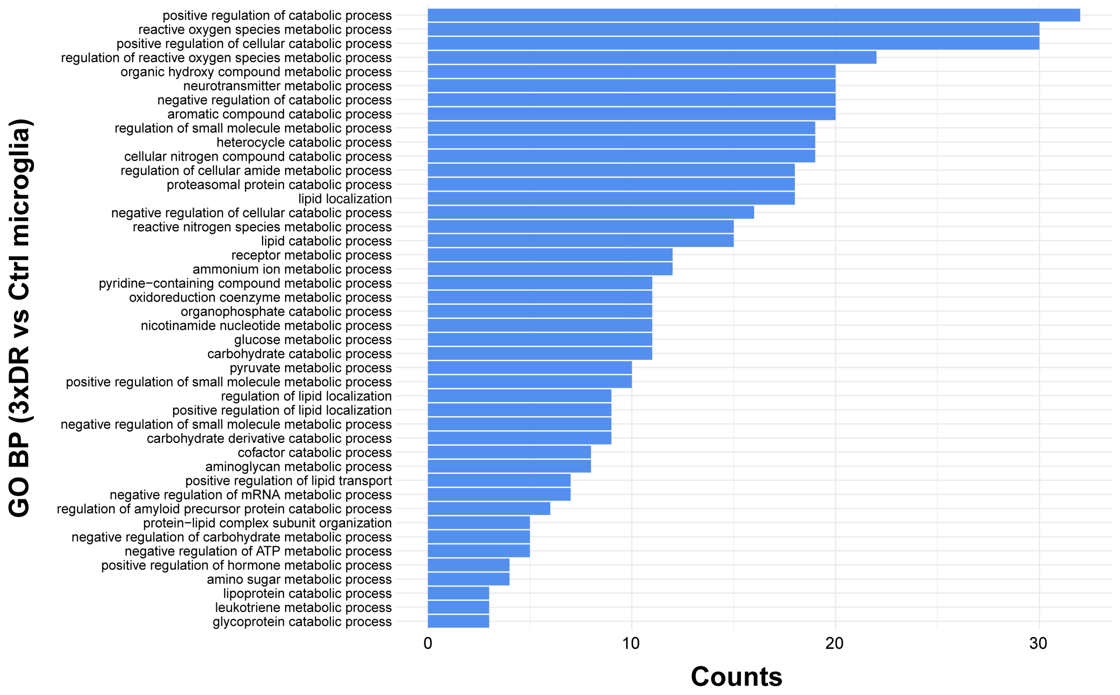

Supplementary Figure 10 Enriched metabolism-related biological processes in 3xDR microglia by Gene Ontology.

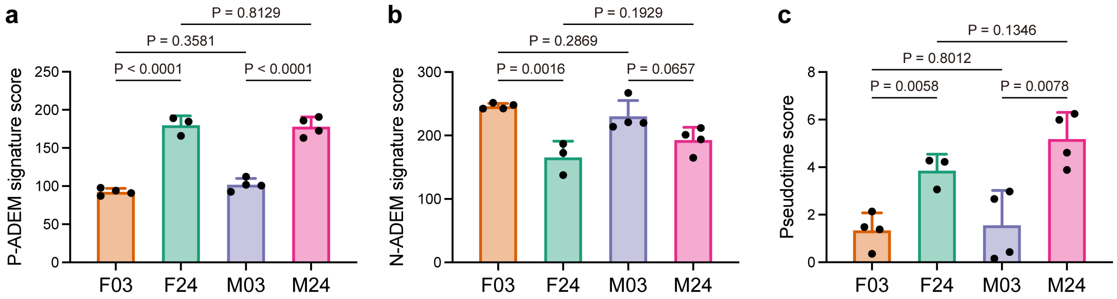

Supplementary Figure 11 Bulk RNA-seq results reveal that both female and male microglia exhibit similar trends in age-related scores.

(a) Both female and male aged microglia exhibit an increased tread in P-ADEM signature score.

(b) Both female and male aged microglia exhibit a decreased tread in N-ADEM signature score.

(c) Both female and male aged microglia exhibit an increased tread in pseudotime score.

Data are presented as mean ± SD.

N = 4, 3, 4 and 4 mice for F03, F24, M03 and M24, respectively. One-way ANOVA with Holm-Šídák's post hoc. F03: female 3-month-old, F24: female 24-month-old, M03: male 3-month-old, M24: male 24-month-old.

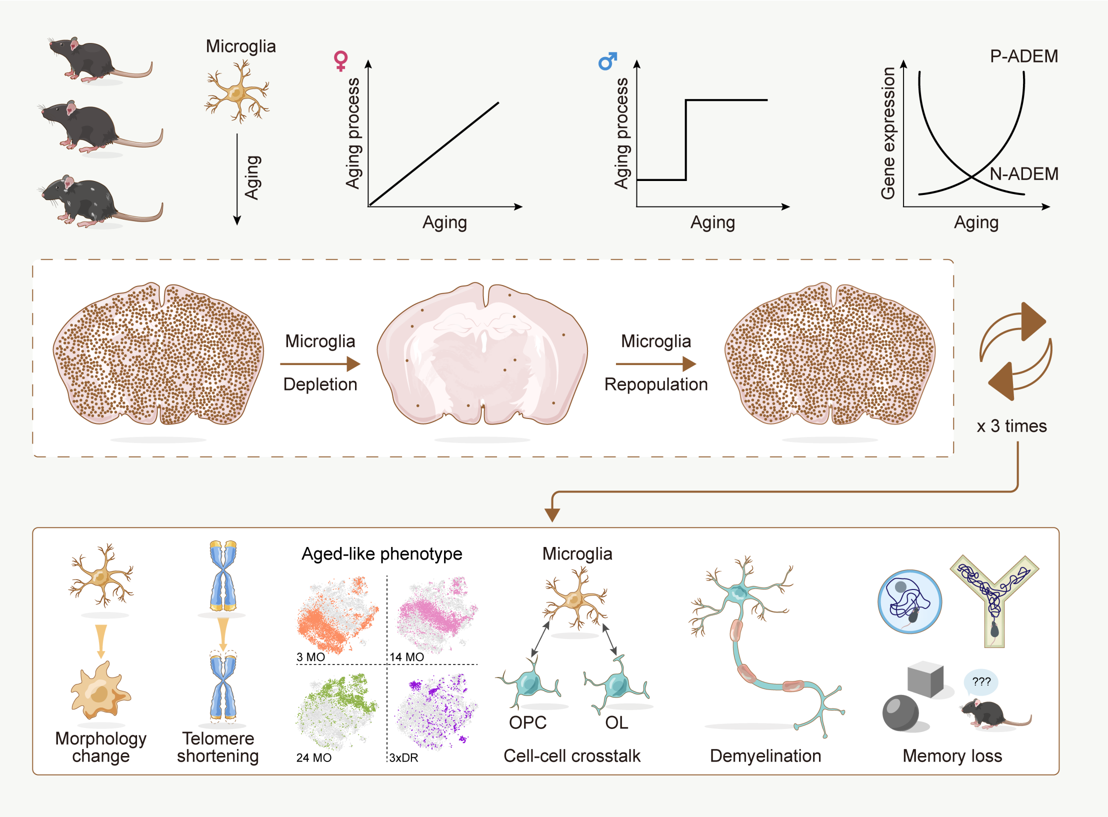

Supplementary Figure 12 Schematic summary of this study.

This figure summarizes the major discovery of this study.
