# Supplementary material for: Transcriptional and epigenetic decoding of the microglial aging process
Source: Nat Aging. 2023 Sep 11;3(10):1288–311. doi: 10.1038/s43587-023-00479-x (PMC10570141; doi:10.1038/s43587-023-00479-x)
Supplement: Supplementary file 2 — Reporting Summary [file 43587_2023_479_MOESM2_ESM.pdf]

nature portfolio

Corresponding author(s): Yanxia Rao and Bo Peng

Last updated by author(s): 2/July/2023

Reporting Summary

Nature Portfolio wishes to improve the reproducibility of the work that we publish. This form provides structure for consistency and transparency in reporting. For further information on Nature Portfolio policies, see our Editorial Policies and the Editorial Policy Checklist.

Statistics

For all statistical analyses, confirm that the following items are present in the figure legend, table legend, main text, or Methods section.

n/a	Confirmed	
☐	☒	The exact sample size (*n*) for each experimental group/condition, given as a discrete number and unit of measurement
☐	☒	A statement on whether measurements were taken from distinct samples or whether the same sample was measured repeatedly
☐	☒	The statistical test(s) used AND whether they are one- or two-sided<br>*Only common tests should be described solely by name; describe more complex techniques in the Methods section.*
☒	☐	A description of all covariates tested
☒	☐	A description of any assumptions or corrections, such as tests of normality and adjustment for multiple comparisons
☐	☒	A full description of the statistical parameters including central tendency (e.g. means) or other basic estimates (e.g. regression coefficient) AND variation (e.g. standard deviation) or associated estimates of uncertainty (e.g. confidence intervals)
☐	☒	For null hypothesis testing, the test statistic (e.g. *F*, *t*, *r*) with confidence intervals, effect sizes, degrees of freedom and *P* value noted<br>*Give P values as exact values whenever suitable.*
☒	☐	For Bayesian analysis, information on the choice of priors and Markov chain Monte Carlo settings
☒	☐	For hierarchical and complex designs, identification of the appropriate level for tests and full reporting of outcomes
☒	☐	Estimates of effect sizes (e.g. Cohen's *d*, Pearson's *r*), indicating how they were calculated

Our web collection on statistics for biologists contains articles on many of the points above.

Software and code

Policy information about availability of computer code

Data collection	scRNA-seq data was collected on Illumina HiSeq 4000 and Illumina HiSeq 6000. Bulk RNA-seq and ATAC-seq data was collected on BGISEQ-500.
Data analysis	1. Fiji 2.9.2 for immunostaining data analysis.<br>2. EthoVision 11.5.1022 for behavior experiments.<br>3. R 3.6.1, HISAT2 2.0.4, Cutadapt 2.8, Bowtie2 2.2.5, RSEM 1.2.12, SOAPnuke 1.5.2, SVA 3.35.2, factoextra 1.0.7, EdgeR 3.28.1, clusterProfiler 3.14.3, HOMER 4.10.0, VennDiagram 1.6.20, pheatmap 1.0.12, EnhancedVolcano 1.4.0, Cell Ranger 2.0.1 or 5.0.0, STAR 2.5.3, Seurat 3.2.0, CellPhoneDB v.2.0, CellChat 1.6.0., Monocle 3 (0.2.2), IGV 2.8.0, Rsubread 2.0.1, ChIPseeker 1.22.1 for sequencing data analysis.<br>4. Prism 8.3.0 for statistic analysis.<br>5. FlowJo 10.4 for FACS data analyses.

For manuscripts utilizing custom algorithms or software that are central to the research but not yet described in published literature, software must be made available to editors and reviewers. We strongly encourage code deposition in a community repository (e.g. GitHub). See the Nature Portfolio guidelines for submitting code & software for further information.

Data

Policy information about availability of data

All manuscripts must include a data availability statement. This statement should provide the following information, where applicable:

- Accession codes, unique identifiers, or web links for publicly available datasets
- A description of any restrictions on data availability
- For clinical datasets or third party data, please ensure that the statement adheres to our policy

Bulk RNA-seq data are available in the Gene Expression Omnibus (GEO) with accession code GSE208386 (reviewer token: idifcyskzbczrop). scRNA-seq data of the microglia of 3-month-old, 14-month-old and 24-month-old PBS- and LPS-treated mice are available in GEO with accession code GSE207932 (reviewer token: gpkvmcuupdezvkn). scRNA-seq data of control and 3xDR microglia are available in GEO with accession code GSE207948 (reviewer token: qvypsiemplwrtcv). scRNA-seq data of brain cells at 3- and 24-month-old are available in GEO with accession code GSE208292 (reviewer token: wtejowywjtapben). scRNA-seq data of control and 3xDR brain cells are available in GEO with accession code GSE226286 (reviewer token: mxmxmkymbhyrfgn). ATAC-seq data are available in GEO with accession code GSE208346 (reviewer token: orgrsywwjxgrxel). Processed data are available in Supplementary Tables 1-7. To disseminate these data to the community, we generated an interactive website for searching the data (http://www.microgliatlas.com).

Human research participants

Policy information about studies involving human research participants and Sex and Gender in Research.

Reporting on sex and gender	N/A
Population characteristics	N/A
Recruitment	N/A
Ethics oversight	N/A

Note that full information on the approval of the study protocol must also be provided in the manuscript.

Field-specific reporting

Please select the one below that is the best fit for your research. If you are not sure, read the appropriate sections before making your selection.

☒ Life sciences ☐ Behavioural & social sciences ☐ Ecological, evolutionary & environmental sciences

For a reference copy of the document with all sections, see nature.com/documents/nr-reporting-summary-flat.pdf

Life sciences study design

All studies must disclose on these points even when the disclosure is negative.

Sample size

For bulk RNA-seq, there are 2 biological replicates in F06_MO and M12_MO group, 3 replicates in F09_MO and F24_MO group, 4 replicates in F03_MO, F12_MO, M03_MO, M06_MO,M09_MO, M16_MO and M24_MO, 5 replicates in F14_MO, F16_MO and M19_MO. For scRNA-seq (except for 3xDR vs control brain cells), each group contains 5 biological replicates (mice). For scRNA-seq of 3xDR vs control brain cells, each group contains 2(3xDR) to 3(Control) biological replicates (mice). For ATAC-seq, we pooled microglia from 5 mice into 2 libraries (2 to 3 mice for each library).
For telomere length measurement, there are 7 mice for each group. For sholl analysis, 8 and 9 mice were analyzed in control and 3xDR group respectively. For senescence index calculation, 8 and 7 mice were measured for control and 3xDR, respectively.
For AXL immunohistochemistry experiments, there are 9 mice used in Ctrl and 3xDR group. For OPN immunostaining, there are 10 mice used in Ctrl and 3xDR group. For MBP immunostaining, there are 9 mice used in Ctrl and 8 used in 3xDR group. For β-gal immunohistochemistry experiments, there are 9 mice used in Ctrl and 3xDR group. For IBA1 immunostaining,4 Tmem119-GFP mice and 8 P2Y12-CreER-GFP mice were measured. For Ki67 and DCX co-immunostaining, N = 7 and 6 mice for control and 3xDR. For NeuN and Pdgfra immunostaining, N = 8 and 7 mice for control and 3xDR group. For CD74 immunostaining, 35 cells in F03 and 49 cells in F24 group were measured. For RNAscope experiment of Spp1 gene, 29 cells in F03 and 31 cells in F24 group were measured. For microglia density analysis in 3xDR process, N = 6 (1xDPL, 2xDPL, 3xDPL, 1xDR and 2xDR) to 7 mice (3xDR) for each group.
For primary microglia siS100a8 knockdown experiment, in the negative control group, N = 5, 3, 3, 3, 3, 3, 3, 5, 3, 3, 5, 3, 4 and 3 biological replicates for S100a8, Ccl3, Ccl4, Ccl5, Ccl12, Cd14, Cxcl13, Ifitm2, Ifitm3, Il1b, Il6, Myd88, Socs3 and Tnf, respectively, in the siS100a8 group, N = 6, 6, 6, 6, 6, 6, 6, 9, 6, 6, 9, 6, 9 and 6 biological replicates for S100a8, Ccl3, Ccl4, Ccl5, Ccl12, Cd14, Cxcl13, Ifitm2, Ifitm3, Il1b, Il6, Myd88, Socs3 and Tnf, respectively. For primary microglia siS100a9 knockdown experiment, in the negative control group, N = 5, 3, 3, 3, 3, 3, 3, 5, 3, 3, 5, 3, 4 and 3 biological replicates for S100a8, Ccl3, Ccl4, Ccl5, Ccl12, Cd14, Cxcl13, Ifitm2, Ifitm3, Il1b, Il6, Myd88, Socs3 and Tnf, respectively, in the siS100a8 group, N = 6, 6, 6, 6, 6, 6, 6, 9, 6, 6, 9, 6, 9 and 6 biological replicates for S100a8, Ccl3, Ccl4, Ccl5, Ccl12, Cd14, Cxcl13, Ifitm2, Ifitm3, Il1b, Il6, Myd88, Socs3 and Tnf, respectively.
For OF and NOR test, N = 12, 10, 11 and 11 mice for young, aged, control and 3xDR groups, respectively. For Y maze test, N = 10 mice for each group. For Morris water maze, N = 10 and 11 mice for control and 3xDR, respectively. For Three-chamber test, N = 8 for control mice and 7 for 3xDR mice.

The detailed sample size is listed in each figure legend.
The statistical approaches were indicated in figure legends. No statistical methods were used to pre-determine sample sizes but our sample sizes are similar to those reported in previous publications1-7. Data distribution was assumed to be normal but this was not formally tested.

References
1. Zhou, T., et al. Microglial debris is cleared by astrocytes via C4b-facilitated phagocytosis and degraded via RUBICON-dependent noncanonical autophagy in mice. Nature communications 13, 6233 (2022).
2. Niu, F., et al. The m6A reader YTHDF2 is a negative regulator for dendrite development and maintenance of retinal ganglion cells. Elife 11, e75827 (2022).
3. Rao, Y., et al. NeuroD1 induces microglial apoptosis and cannot induce microglia-to-neuron cross-lineage reprogramming. Neuron 109, 4094-4108.e4095 (2021).
4. Xu, Z., et al. Efficient strategies for microglia replacement in the central nervous system. Cell reports 32, 108041 (2020).
5. Huang, Y., et al. Dual extra-retinal origins of microglia in the model of retinal microglia repopulation. Cell Discov 4, 9 (2018).
6. Huang, Y., et al. Repopulated microglia are solely derived from the proliferation of residual microglia after acute depletion. Nature neuroscience 21, 530-540 (2018).
7. Peng, B., et al. Suppression of microglial activation is neuroprotective in a mouse model of human retinitis pigmentosa. J Neurosci 34, 8139-8150 (2014).

Data exclusions

Exclusion criteria for experimental data points were sickness or death of animals during the experimental period. Or low quality sequencing results were excluded. No outliers were excluded in this manuscript.

Replication

For each in vivo experimental condition, at least at least 5 animals were used per conditions. All attempts at replication were successful expect for the failures due to sickness and death of animals or technical faults during the experimental period. Or low quality sequencing results were excluded.

Randomization

Mice were randomized from each group if possible.

Blinding

Behavioral examinations and sequencing experiments were conducted blind. Sequencing data analysis were not performed blind to the conditions of the experiments. Because there were no needs to be blinded for the sequencing data analyses.

Reporting for specific materials, systems and methods

We require information from authors about some types of materials, experimental systems and methods used in many studies. Here, indicate whether each material, system or method listed is relevant to your study. If you are not sure if a list item applies to your research, read the appropriate section before selecting a response.

Materials & experimental systems

n/a	Involved in the study	
☐	☒	Antibodies
☒	☐	Eukaryotic cell lines
☒	☐	Palaeontology and archaeology
☐	☒	Animals and other organisms
☒	☐	Clinical data
☒	☐	Dual use research of concern

Methods

n/a	Involved in the study	
☒	☐	ChIP-seq
☐	☒	Flow cytometry
☒	☐	MRI-based neuroimaging

Antibodies

Antibodies used

The primary antibodies used in this study included rabbit anti-IBA1 (1:500, Wako, cat: 019-19741, lot: CAJ3125, SKM6526 and LEQ2171), goat anti-IBA1 (1:500, Abcam, cat: Ab5076, lot: GR3381291-3 and GR3365012-2), goat anti-Osteopontin/OPN (1:500, R&D system, cat: AF808, lot: BDO0720111), goat anti-AXL (1:200, R&D system, cat: AF854, lot: CTC0220081), rabbit anti-beta galactosidase (1:2000, Invitrogen, cat: A-11132, lot: 2304273), rabbit anti-Ki67 (1:250, Invitrogen, cat: MA5-14520, lot: VB2941291, VE3003591), rat anti-Ki67 (1:1000, Invitrogen, cat: 14-5698-82, lot: 2496198), rabbit anti-DCX (1:200, Abcam, cat: ab18723, lot: GR3274138-3), rabbit anti-PDGFRα (1:500, Cell Signaling, cat: 3164S, lot: 02/2020-6), rabbit anti-NeuN (1:500, Abcam, cat: ab177487, lot: GR3275122-6), and rabbit anti-MBP (1:500, Abcam, cat: ab218011, lot: GR3299139-18) (Supplementary Table 8).

The secondary antibodies used in this study included AF647 donkey anti-goat (Jackson ImmunoResearch, cat: 705-605-003, lot:147708), AF488 donkey anti-chicken (Jackson ImmunoResearch, cat: 703-545-155, lot:147805), AF488 donkey anti-mouse (Jackson ImmunoResearch, cat: 715-545-150, lot:146643), AF488 donkey anti-goat (Jackson ImmunoResearch, cat: 705-545-003, lot:145270), and Cy3 donkey anti-rabbit (Jackson ImmunoResearch, cat: 711-165-152, lot:145020) (Supplementary Table 8).

Validation

The validation data of each antibody are listed in the websites of corresponding manufactures.

For primary antibody:
rabbit anti-IBA1 (1:500, Wako, cat: 019-19741, lot: CAJ3125, SKM6526 and LEQ2171) has been validated for use in IF and IHC, as stated on the product page and has been referenced in at lesat 8 papers (https://labchem-wako.fujifilm.com/us/product/detail/W01W0101-1974.html).

goat anti-IBA1 (1:500, Abcam, cat: Ab5076, lot: GR3381291-3 and GR3365012-2) has been validated for use in IHC-P, WB, as stated

on the product page and has been referenced in 1145 papers (https://www.abcam.com/products/primary-antibodies/iba1-antibody-ab5076.html).

goat anti-Osteopontin/OPN (1:500, R&D system, cat: AF808, lot: BDO0720111) has been validated for use in IHC, WB and ICC, as stated on the product page and has been referenced in 120 papers (https://www.rndsystems.com/cn/products/mouse-osteopontin-opn-antibody_af808).

goat anti-AXL (1:200, R&D system, cat: AF854, lot: CTC0220081) has been validated for use in IHC, WB, as stated on the product page and has been referenced in 24 papers(https://www.rndsystems.com/cn/products/mouse-axl-antibody_af854).

rabbit anti-beta galactosidase (1:2000, Invitrogen, cat: A-11132, lot: 2304273) has been validated for use in IHC, WB, ICC/IF, ELISA, ChIP, as stated on the product page and has been referenced in 95 papers (https://www.thermofisher.cn/cn/zh/antibody/product/beta-Galactosidase-Antibody-Polyclonal/A-11132).

rabbit anti-Ki67 (1:250, Invitrogen, cat: MA5-14520, lot: VB2941291, VE3003591) has been validated for use in IHC, WB, ICC/IF, Flow, FN, as stated on the product page and has been referenced in 742 papers (https://www.thermofisher.cn/cn/zh/antibody/product/Ki-67-Antibody-clone-SP6-Recombinant-Monoclonal/MA5-14520).

rat anti-Ki67 (1:1000, Invitrogen, cat: 14-5698-82, lot: 2496198) has been validated for use in IHC, WB, ICC/IF, Flow, FN, as stated on the product page and has been referenced in 305 papers (https://www.thermofisher.cn/cn/zh/antibody/product/Ki-67-Antibody-clone-SolA15-Monoclonal/14-5698-82).

rabbit anti-DCX (1:200, Abcam, cat: ab18723, lot: GR3274138-3) has been validated for use in WB, IHC-FoFr, ICC/IF, IHC-Fr, IHC-P, as stated on the product page and has been referenced in 400 papers (https://www.abcam.com/products/primary-antibodies/doublecortin-antibody-ab18723.html).

rabbit anti-PDGFRα (1:500, Cell Signaling, cat: 3164S, lot: 02/2020-6) has been validated for use in WB, ICC/IF, IP, as stated on the product page and has been referenced in 127 papers (https://www.cellsignal.com/products/primary-antibodies/pdgf-receptor-a-antibody/3164).

rabbit anti-NeuN (1:500, Abcam, cat: ab177487, lot: GR3275122-6) has been validated for use in WB, ICC/IF, Flow Cyt, as stated on the product page and has been referenced in 695 papers (https://www.abcam.com/products/primary-antibodies/neun-antibody-epr12763-neuronal-marker-ab177487.html).

rabbit anti-MBP (1:500, Abcam, cat: ab218011, lot: GR3299139-18) has been validated for use in WB, IHC-P, IHC-Fr, as stated on the product page and has been referenced in 18 papers (https://www.abcam.com/products/primary-antibodies/myelin-basic-protein-antibody-epr21188-ab218011.html).

For secondary antibody:
AF647 donkey anti-goat (Jackson ImmunoResearch, cat: 705-605-003, lot:147708)has been validated as stated on the product page and has been referenced in 52 papers (https://www.jacksonimmuno.com/catalog/products/705-605-003).

AF488 donkey anti-chicken (Jackson ImmunoResearch, cat: 703-545-155, lot:147805) has been validated as stated on the product page and has been referenced in 816 papers (https://www.jacksonimmuno.com/catalog/products/703-545-155).

AF488 donkey anti-mouse (Jackson ImmunoResearch, cat: 715-545-150, lot:146643) has been validated as stated on the product page and has been referenced in 605 papers (https://www.jacksonimmuno.com/catalog/products/715-545-150).

AF488 donkey anti-goat (Jackson ImmunoResearch, cat: 705-545-003, lot:145270)has been validated as stated on the product page and has been referenced in 137 papers (https://www.jacksonimmuno.com/catalog/products/705-545-003).

Cy3 donkey anti-rabbit (Jackson ImmunoResearch, cat: 711-165-152, lot:145020) has been validated as stated on the product page and has been referenced in 1702 papers (https://www.jacksonimmuno.com/catalog/products/711-165-152).

Animals and other research organisms

Policy information about studies involving animals; ARRIVE guidelines recommended for reporting animal research, and Sex and Gender in Research

Laboratory animals	C57BL/6J mice were either purchased from SPF (Beijing) Biotechnology Co., Ltd or donated by Prof. Zhihui Huang at Hangzhou Normal University. P2Y12-CreER-GFP mice (P2ry12-p2A-CreER-p2A-EGFP) were donated by Prof. Jiyun Peng at Nanchang University, histological analysis were performed at 4 month old. TMEM119-GFP mice (C57BL/6-Tmem119em2(EGFP)Gfng/J, Stock #: 31823) was purchased from Jackson Lab, histological analysis were performed at 4-month-old. All mice were housed in the Animal Facility at Department of Laboratory Animal Science at Fudan University or Shenzhen Institute of Advanced Technology at Chinese Academy of Sciences under a 12-hour light/dark cycle with food and water ad libitum, room temperature was maintained at 21-25 degree centigrade, humidity was maintained at 40%-65% .
Wild animals	This study did not involve wild animals.
Reporting on sex	The animal sex was described in the text and/or figure legend.
Field-collected samples	This study did not involve field-collected samples.
Ethics oversight	All animal experiments were conducted in accordance with the guidelines of the Institutional Animal Care and Use Committee of

Ethics oversight	Department of Laboratory Animal Science at Fudan University (202009001S, 202110005S and 2021JS-ITBR-002) and Institutional Animal Care and Use Committee at Shenzhen Institute of Advanced Technology, Chinese Academy of Sciences (SIAT-IACUC-190312-YGS-PB-A0576-01).

Note that full information on the approval of the study protocol must also be provided in the manuscript.

Flow Cytometry

Plots

Confirm that:

- [x] The axis labels state the marker and fluorochrome used (e.g. CD4-FITC).
- [x] The axis scales are clearly visible. Include numbers along axes only for bottom left plot of group (a 'group' is an analysis of identical markers).
- [x] All plots are contour plots with outliers or pseudocolor plots.
- [ ] A numerical value for number of cells or percentage (with statistics) is provided.

Methodology

Sample preparation	For the brain cell scRNA-seq, brains without cerebellum were minced into pieces and then dissociated in 8 U/mL papain lysis buffer containing 125 U/mL DNase I at 37 °C for 20 minutes with mild shaking. This process was then terminated by adding a 10% ovomucoid in L15 culture medium. After that, cell clusters were removed by filtering through a 70 μm nylon strainer (Falcon). Myelin and cell debris were removed through the density gradient centrifugation in 37% Percol (Solarbio Life Science). Next, brain cells were thoroughly rinsed by EDTA-free FACS buffer (0.5% BSA in DPBS) before library preparation.<br><br>Microglia from young, middle-aged and old mice were sorted by FACS as we previously described28,74. Briefly, the mixed brain cells were resuspended by FACS buffer. Then, mixed cells were stained with antibodies against CD11b (1:100, clone M1/70, BD Pharmingen, cat: 557657) and CD45 (1:100, clone 30-F11, BD Pharmingen, cat: 553080) in FACS buffer for 30 min in ice. Dead cells were labeled by 7-AAD (1:80, BD Pharmingen, cat: 559925). Then, CD11b+ CD45low 7-ADD- microglia were collected by FACSAria III cell sorted (BD Biosciences). Harvested cells were then used for scRNA-seq, bulk RNA-seq and ATAC-seq.
Instrument	FACSAria III cell sorted (BD Biosciences)
Software	Cell sorter was controlled by Summit. FACS data were analyzed by FlowJo 10.4.
Cell population abundance	After removal the doublelets and cell debris by FSC/SSC, CD11b+ CD45low 7-AAD- cells were sorted for subsequent analysis.
Gating strategy	After removal the doublelets and cell debris by FSC/SSC, CD11b+ CD45low 7-AAD- cells were sorted for subsequent analysis.

- [x] Tick this box to confirm that a figure exemplifying the gating strategy is provided in the Supplementary Information.
